# Cold Temperature Direct Air CO_2_ Capture with Amine-Loaded Metal–Organic Framework Monoliths

**DOI:** 10.1021/acsami.3c13528

**Published:** 2023-12-18

**Authors:** Yuxiang Wang, Guanhe Rim, MinGyu Song, Hannah E. Holmes, Christopher W. Jones, Ryan P. Lively

**Affiliations:** School of Chemical & Biomolecular Engineering, Georgia Institute of Technology, 311 Ferst Dr., Atlanta, Georgia 30332, United States

**Keywords:** 3D printing, carbon capture, direct air capture, metal−organic frameworks, subambient conditions

## Abstract

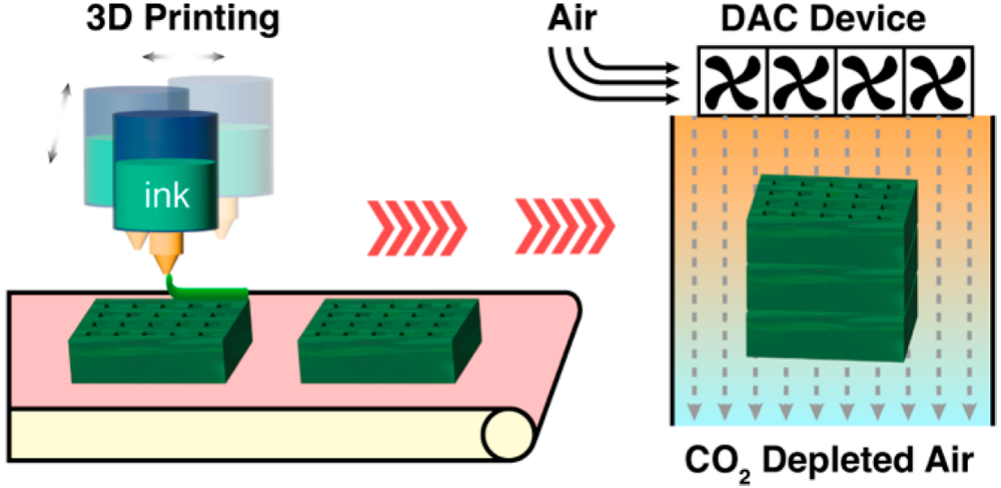

Zeolites, silica-supported amines, and metal–organic frameworks (MOFs) have been demonstrated as promising adsorbents for direct air CO_2_ capture (DAC), but the shaping and structuring of these materials into sorbent modules for practical processes have been inadequately investigated compared to the extensive research on powder materials. Furthermore, there have been relatively few studies reporting the DAC performance of sorbent contactors under cold, subambient conditions (temperatures below 20 °C). In this work, we demonstrate the successful fabrication of adsorbent monoliths composed of cellulose acetate (CA) and adsorbent particles such as zeolite 13X and MOF MIL-101(Cr) by a 3D printing technique: solution-based additive manufacturing (SBAM). These monoliths feature interpenetrated macroporous polymeric frameworks in which microcrystals of zeolite 13X or MIL-101(Cr) are evenly distributed, highlighting the versatility of SBAM in fabricating monoliths containing sorbents with different particle sizes and density. Branched poly(ethylenimine) (PEI) is successfully loaded into the CA/MIL-101(Cr) monoliths to impart CO_2_ uptakes of 1.05 mmol g_monolith_^–1^ at −20 °C and 400 ppm of CO_2_. Kinetic analysis shows that the CO_2_ sorption kinetics of PEI-loaded MIL-101(Cr) sorbents are not compromised in the monoliths compared to the powder sorbents. Importantly, these monoliths exhibit promising working capacities (0.95 mmol g_monolith_^–1^) over 14 temperature swing cycles with a moderate regeneration temperature of 60 °C. Dynamic breakthrough experiments at 25 °C under dry conditions reveal a CO_2_ uptake capacity of 0.60 mmol g_monolith_^–1^, which further increases to 1.05 and 1.43 mmol g_monolith_^–1^ at −20 °C under dry and humid (70% relative humidity) conditions, respectively. Our work showcases the successful implementation of SBAM in making DAC sorbent monoliths with notable CO_2_ capture performance over a wide range of sorption temperatures, suggesting that SBAM can enable the preparation of efficient sorbent contactors in various form factors for other important chemical separations.

## 1 Introduction

Direct air capture (DAC) of CO_2_ from the atmosphere based on adsorption processes has garnered tremendous interest as a potentially scalable negative emissions technology. A large number of publications have reported the DAC performance of adsorbents at ambient conditions (i.e., temperatures >20 °C), but there has been only limited investigation of lower temperatures.^1^ Compared to DAC under ambient conditions, DAC at colder temperatures may allow the usage of physisorbents with lower CO_2_ heat of adsorption and hence enable DAC processes with lower energy consumption. In addition, the lower absolute humidity at colder temperatures could potentially reduce the energy consumed for water desorption, and it may be advantageous to perform DAC at low temperatures to reduce the oxidative degradation rate of PEI.^2,3^ This research gap hampers the rapid development and deployment of adsorption-based DAC processes in many areas of the world where the annual average temperature is below the typical temperature of research laboratories (20–30 °C). Song et al. investigated the potential of using commercially available zeolite adsorbents for DAC under subambient conditions.^4^ It was found that a predrying step before adsorption could be considered at cold temperatures, whereas this approach would be cost prohibitive at higher temperatures where water vapor content in the air can be much higher.

Adsorbents with amine functionalities that are covalently grafted to or physically entrapped in the pores of support materials, such as silica, cellulose aerogel, or metal–organic frameworks (MOFs), have demonstrated encouraging DAC performance under both dry and humid conditions at ambient laboratory temperatures. However, their performance at subambient conditions has been underexplored.^5−11^ Rim et al. studied the DAC performance of supported poly(ethylenimine) (PEI) and tetraethylenepentamine (TEPA) in MIL-101(Cr) under subambient conditions.^12^ When the amine loading is moderate, the amine–CO_2_ interactions have moderate enthalpies of adsorption, akin to weak chemical interactions, providing stable working capacities (up to 0.75 mmol g^–1^) with narrow temperature swing windows (e.g., – 20 to 25 °C). Similar to previous works focusing on ambient temperatures or above,^13^ enhancement of the subambient DAC performance of amine-impregnated MIL-101(Cr) was also observed under humid conditions. A recent study showed that the high surface area to pore volume ratio of MIL-101(Cr) results in weak chemisorption (the formation of carbamic acid) of CO_2_ in MIL-101(Cr)-supported TEPA, which requires less energy consumption for CO_2_ desorption compared to the case of strong chemisorption of CO_2_ (the formation of carbamate).^14^ These results suggest the intriguing possibility of using amine-based adsorbents for DAC under cold conditions with lower energy consumption relative to hot and humid climates.

Because of the low concentration of CO_2_ in air, large quantities of air must be processed to capture significant amounts of CO_2_. For this reason, it is important to translate the adsorbents from the initially studied powder form into other geometries and structures to achieve low pressure drops along the sorption bed without significantly increasing mass transfer resistances or compromising the uptake capacities. Prior works have explored a variety of forms of adsorbents including pellets,^15−17^ fibers,^18^ flat sheets,^19^ and monoliths for DAC.^20^ However, all known structured DAC contactor studies focus on ambient or warmer testing conditions.

Recently, additive manufacturing, or 3D printing, has emerged as a nascent technology to fabricate adsorption contactors for a variety of separation applications.^21^ Direct ink writing (DIW) is the most common 3D printing approach that has been used to prepare monolithic sorbent structures, where ink containing the adsorbent powder is continuously extruded out of a printer head (nozzle) and deposited using precise spatial coordinates predetermined by 3D printing programs. Compared with conventional shaping methods such as pelletizing, molding, and extrusion, 3D printing can potentially afford better spatial manufacturing resolutions to allow the design and manufacture of novel sorption contactors with complex engineered geometries. If carefully designed, such geometries can potentially enhance the mass and heat transfer performance of these contactors, as suggested by computational simulations.^22^

To date, a wide variety of adsorbent particles including porous carbons,^23,24^ zeolites,^25−27^ MOFs,^28−31^ and covalent–organic frameworks (COFs)^32^ have been formulated into printable inks for 3D printing of monolithic structures for chemical separations. For example, Pereira et al. used 3D printing to fabricate a monolith containing zeolite 13X and carbon black particles.^25^ Because of the short distance between carbon and zeolite 13X, zeolite 13X can be quickly regenerated by resistance heating of carbon particles when a voltage is applied to the contactor, which may allow for higher energy efficiency compared to typical sorbent regeneration methods based on heat exchange liquids or vapors (e.g., indirect steam stripping). The Rezaei group explored a series of ink formulations for 3D printing based on water, clay, poly (vinyl alcohol) (PVA), and porous adsorbents.^26^ This formulation has been used to fabricate monoliths of zeolites and MOFs for not only gas sorption but also heterogeneous catalysis.^28,30,33,34^ For example, Lawson et al. used this ink system to prepare MIL-101(Cr) monoliths and impregnate amines in these monoliths for CO_2_ removal in an enclosed environment.^30^ More recently, this ink formulation was adopted to fabricate monoliths containing two types of nanoparticles: Fe_3_O_4_ and Ni-MOF-74.^35^ Induction heating of the Fe_3_O_4_ nanoparticles enabled the rapid heating and regeneration of the adsorbent monolith. In another example, Grande et al. formulated a nonaqueous ink containing UTSA-16, hydroxypropyl cellulose, boehmite AlO(OH), and isopropyl alcohol with suitable rheological properties to fabricate UTSA-16 monoliths for CO_2_ capture.^31^*In situ* synchrotron XRD-CT data were collected to reveal insights into the spatial and temporal evolution of UTSA-16 in the monoliths during CO_2_ sorption. Together, these examples showcase the versatility of these 3D printing methods to fabricate monoliths with complicated compositions. Despite the high sorbent loadings, some monoliths exhibited little interparticle porosity, which could be a source of the observed mass transfer resistances.^30^

Solution-based additive manufacturing (SBAM), another method of DIW 3D printing, utilizes phase separation of polymer solutions to generate macropores in sorbent monoliths.^36^ In this approach, viscous polymeric dopes composed of polymers, solvent, and nonsolvent are used as the inks for printing. Once the ink is extruded out of the nozzle and deposited on a substrate, the evaporation of volatile solvents leads to spinodal decomposition of the deposited polymeric filaments, which not only increases the storage modulus of the filaments for better preservation of filament shape but also affords interpenetrated polymer-lean and polymer-rich phases within the filaments. The polymer-lean phase can be subsequently removed by solvent exchange after printing to generate macropores that are beneficial for rapid mass transfer inside of the filaments. Zhang et al. showed that SBAM is applicable to printing a variety of polymers including cellulose acetate (CA), Matrimid, and polymers of intrinsic porosity PIM-1.^36,37^ PIM-1 was fabricated into air contactors by SBAM with superior mass transfer efficiency and toluene uptake capacities compared to those of contactors using PIM-1 in the form of pellets and fibers. In addition to solvent evaporation, spinodal decomposition could also be triggered by the diffusion of nonsolvent vapor into the deposited polymer filaments. For example, Xu et al. controlled the internal porosity and layer adhesion of printed filaments composed of a mixture of 2-pyrrolidinone, poly(sulfone), poly(styrene)-*block*-poly(acrylic acid), and carbon nanotubes by carefully modulating the humidity level in the printing environment.^38^ The 3D printed structured sorbents were subsequently modified by poly(ethylenimine) (PEI) and terpyridine for the efficient removal of metal ions from water under dynamic flow conditions.

To date, DIW using an ink based on polymeric solutions has not been employed to fabricate structures that contain high loadings of adsorbents for chemical separations. The incorporation of solid particles will change the rheological properties of the ink, and solid particles may agglomerate to clog the printer nozzles if these particles are not well dispersed before printing.^39^ In addition, because porous adsorbent particles typically comprise greater volume fractions in the ink than nonporous particles at the same weight loading, the changes in rheological properties (e.g., viscosity) of the ink brought on by sorbents will be much more significant compared to the changes caused by adding nonporous particles. Therefore, it is challenging to print structures with high weight loadings of adsorbents, which is important for minimizing any decline in separation performance due to the introduction of dead weight into the printed structures. In this work, we describe the utilization of SBAM to prepare sorption contactors for DAC and explore the sorption performance of the contactors under primarily subambient conditions. Cellulose acetate (CA) is used as the macroporous support polymer for these contactors, and microcrystals of zeolite 13X and MOF MIL-101(Cr) were distributed evenly within this porous polymer matrix. We show that the gravimetric loading of adsorbents can be as high as 70 wt % in these cases, and the DAC performance of amine-loaded MIL-101(Cr) sorbents under subambient conditions is well preserved in the polymeric frameworks.

## 2 Experimental Methods

### 2.1 Materials

Chromium(III) nitrate nonahydrate Cr(NO_3_)_3_·9H_2_O (99%), dimethylformamide (DMF, ACS grade), dimethylacetamide (DMAc, ACS grade), cellulose acetate (CA, *M*_n_ ∼ 50000 by GPC, 39.7 wt % acetyl), and branched poly(ethylenimine) (PEI) (*M*_w_ 800 by MS) were purchased from Sigma-Aldrich. Terephthalic acid (H_2_BDC) was purchased from Acros Chemicals. Methanol (ACS grade), hexane (ACS grade), and acetone (ACS grade) were purchased from BDH Chemicals. Hexane (HPLC grade) was purchased from Fisher Scientific. Cylinders of N_2_ (99.999%), bone dry CO_2_ (99.9%), He (99.999%), and 400 ppm of CO_2_ balanced in N_2_ or He were purchased from Airgas.

### 2.2 Synthesis of MIL-101(Cr)

MIL-101(Cr) was synthesized hydrothermally based on the recipe from the literature.^40^ First, 64 g of Cr(NO_3_)_3_·9H_2_O, 27.1 g of H_2_BDC, and 160 mL of HNO_3_ aqueous solution (1 N) were added to 640 mL of deionized (DI) water. The mixture was stirred for 0.5 h followed with sonication in a water bath for 0.5 h. The mixture was subsequently transferred to a 2 L Teflon-lined autoclave and heated at 200 °C for 16 h, followed by slow cooling to room temperature. After the synthesis, large colorless needlelike crystals were removed, and the dark green powder was collected using a centrifuge. The dark green powder was then sequentially washed with DMF (0.9 L, three times), MeOH (0.9 L, twice), and acetone (0.9 L, once). Each washing step lasted for 1 day. The resulting product powders were dried under high vacuum (about 10 mTorr) at 120 °C overnight for further analysis and monolith preparation.

### 2.3 Preparation of Solution-Based Additive Manufacturing (SBAM) Inks for Printing MIL-101(Cr) Monoliths

A typical procedure to prepare the SBAM ink for printing MIL-101(Cr) monoliths with 60 wt % sorbent loading is as follows. First, MIL-101(Cr) powder was activated at 120 °C under vacuum overnight to remove residual solvents in the pores. After activation, the powder was sealed in a jar containing a mixture of DMAc, acetone, and H_2_O with the same compositions as the SBAM ink for 7 days to saturate the pores with solvent vapor. The vapor loading in MIL-101 was determined by thermogravimetric analysis (TGA). Second, a stock solution of acetone (30.8 wt %), DMAc (46.3 wt %), and DI H_2_O (22.9 wt %) was prepared, and 0.3 g of CA was dissolved in 2.16 g of the stock solution to prepare a prime dope. Third, vapor-saturated MIL-101(Cr) (2.2 g, contains 50 wt % vapor of the mixed solvent) was dispersed in 8.42 g of the stock solution by sonication in a water bath for 1.5 h before combining this dispersion dope with the prime dope. More vapor-saturated MIL-101(Cr) (2.4 g) was added to the mixture under stirring, and the mixture was further sonicated in a water bath for 1.5 h and homogenized by the Branson 450 digital sonifier with an output of 20% amplitude for a sonication time of 2 min 20 s (20 s pulse with 20 s interval). Last, the remaining CA (1.2 g) was added, and the vial containing the final mixture was subsequently put on a roller under an infrared lamp for at least 3 days to homogenize the ink before SBAM.

### 2.4 Fabrication of Sorbent Monoliths by SBAM

**Figure 1:**
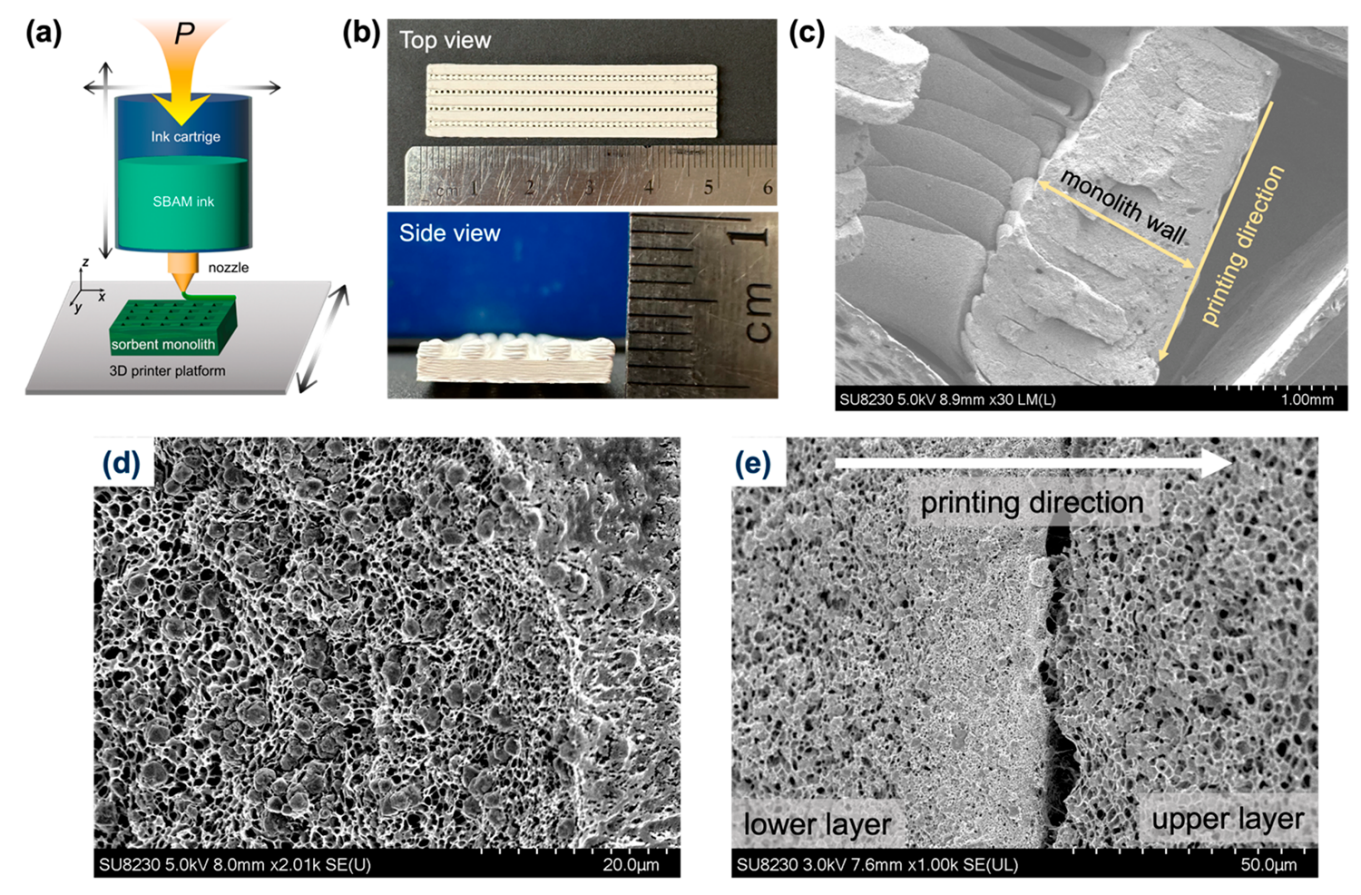
(a) Schematic illustration of the SBAM experimental setup. (b) Pictures of a typical CA/13X monolith fabricated by SBAM from the top (top) and side (bottom) perspectives. (c) Low-magnification cross-sectional SEM image of a CA/13X monolith showing good layer adhesion. (d) A magnified cross-sectional SEM image showing evenly distributed zeolite 13X crystals in the CA matrix of a CA/13X monolith. (e) A cross-sectional SEM image showing the nonuniform pore size distribution at the interface between lower and upper layers.

The structures of the sorbent monoliths were typically designed by Fusion 360. The structural files in STL format were imported into Cura, a 3D printing software of Ultimaker, and converted to G-codes for the control of the 3D printing process. G-codes could also be generated by a Python program that uses structural parameters of monoliths, such as channel widths and monolith heights, as inputs. The SBAM 3D printer was modified from a commercial Cartesian 3D printer Creality CR-10 Max (Figure 1a). The SBAM ink was extruded from the nozzle by N_2_ (69–90 kPa) and deposited on the platform of the 3D printer, during which the nozzle and the platform were not heated (∼23 °C). N_2_ pressure and the gap between the printer nozzle and the platform were carefully controlled to facilitate good adhesion between different layers of filaments. The *x*–*y* translation speed of the nozzle was set to 1 cm s^–1^ during printing. After printing, the monolith was immersed in DI H_2_O for 3 days (water refreshed every day) to achieve complete phase inversion. The monolith was further immersed in methanol and hexane (ACS grade) each for 3 days during which solvent was refreshed every day.

### 2.5 Amine Loading into CA/MIL-101(Cr) Monoliths

The dual-solvent PEI loading method was adopted from the literature with minor modifications to maximize the driving force for infusion of PEI into the pores of MIL-101(Cr).^41^ A typical procedure for loading PEI into CA/MIL-101(Cr) monoliths is described below. CA/MIL-101(Cr) monoliths were first activated at 100 °C under a vacuum overnight to remove residual solvents in the pores. After cooling to room temperature, the monoliths (0.95 g) were placed on a holder in VWR straight sided jars (Scheme S1), to which 224 mL of hexane (HPLC grade) was added. After stirring for 5 min, 9.4 g of 33 wt % PEI/MeOH solution was added dropwise into the hexane solution under vigorous stirring. The monoliths were taken out of the solution after 24 h and dried in a fume hood overnight. The monoliths were then put in a 50 mL beaker, washed by MeOH (24 × 3 mL, 5 min for each washing step, during which the beaker was gently shaken), and dried in a vacuum at room temperature before further characterizations. The amounts of the PEI/MeOH solution were varied in the PEI loading step to tune the PEI loadings in CA/MIL-101(Cr) monoliths.

### 2.6 CO_2_ Adsorption Measurements

The equilibrium CO_2_ uptake capacities of CA/MIL-101/PEI monoliths were measured volumetrically under dry ambient (25 °C) and subambient (− 20 °C) conditions using a surface area and porosity (SAP) system (autosorb iQ/Quantachrome). About 100 mg of the monolith samples was activated at 110 °C under vacuum for 3 h before measuring CO_2_ adsorption capacities. During measurement, CO_2_ is automatically dosed into the sample cells, and the cell pressures were checked every 1 min until the pressure in the cell was within the P tolerance (regulated by the tolerance value “0” to ensure the tightest match between the desired and achieved relative pressures).

Because the SAP system does not provide information about CO_2_ uptake kinetics, the CO_2_ uptake profiles of CA/MIL-101/PEI monoliths were also gravimetrically measured with a TGA/differential scanning calorimetry (DSC) system (STA 449 F3 Jupiter/NETZSCH) under dry conditions at −20 and 25 °C. About 20 mg of the sample was first activated at 110 °C under a He flow (90 mL min^–1^) for 3 h, followed by thermal equilibration under adsorption temperature conditions (−20 or 25 °C). The sample was then exposed to 400 ppm of CO_2_ balanced in He (90 mL min^–1^) for 12 h. The CO_2_ uptake profiles of CA/13X monoliths at 50 kPa of CO_2_ partial pressure and 30 °C were gravimetrically measured with a TGA Q550 from TA Instruments. About 25 mg of the CA/13X monolith sample was activated at 150 °C for 2 h under a N_2_ flow (100 mL min^–1^), followed by thermal equilibration under adsorption temperature conditions (30 °C). The sample was then exposed to 50% CO_2_ balanced in N_2_ (20 mL min^–1^ in total) for 2 h.

Temperature swing adsorption–desorption cyclic tests were performed for up to 14 cycles with the TGA/DSC system. The CO_2_ adsorption step under the 400 ppm of CO_2_ balanced in He gas stream (90 mL min^–1^) at −20 °C and the regeneration step under the He gas stream (90 mL min^–1^) at 60 °C were performed for 2 h each.

### 2.7 CO_2_ Temperature-Programmed Desorption (TPD)

TPD experiments were performed by using the TGA/DSC system. After 400 ppm of CO_2_ adsorption with the powder sorbents for 12 h at −20 °C, the inlet gas flow was changed to pure He, and the TGA/DSC chamber was purged for 1 h at the adsorption temperature condition. The chamber temperature was then slowly increased at a rate of 0.5 °C min^–1^ to 110 °C to desorb CO_2_ from the powder sorbents. During the entire process, the concentrations of CO_2_ and H_2_O of the outlet gas stream were continuously measured by an infrared analyzer LI-COR LI-850 CO_2_/H_2_O gas analyzer to deconvolute the H_2_O and CO_2_ desorption profiles.

### 2.8 Breakthrough Experiments

A schematic illustration of the setup for breakthrough experiments is shown in Scheme S2. Before breakthrough experiments, the bed of the CA/MIL-101/PEI monoliths was purged by 200 sccm of dry N_2_ at 90 °C for 12 h. After activation, the bed was submerged in a bath of a mixture of ethylene glycol and water at predetermined temperatures for at least 0.5 h before starting the breakthrough experiments. For breakthrough experiments under dry conditions, a stream of 400 ppm of CO_2_ balanced in N_2_ was introduced into the bed, and the concentrations of CO_2_ and H_2_O at the outlet of the bed were recorded by a LI-COR LI-850 CO_2_/H_2_O gas analyzer. For breakthrough experiments under wet conditions, the relative humidity of the feed gas was regulated by a LI-COR LI-650 dew point generator. More details about the custom-built fixed bed system and analysis of the breakthrough experiments are available in the Supporting Information.

## 3 Results and Discussion

### 3.1 Fabrication of Zeolite 13X Monoliths via SBAM

Cellulose acetate (CA) was selected as the polymer component of the SBAM printing ink for several reasons. First, CA is readily available and affordable for the potential mass production of sorbent monoliths. Second, the abundant polar functional groups of CA can strengthen the interactions between CA and sorbent particles with polar surfaces, which can hinder loss of sorbent particles from the printed monoliths in postprinting modification steps such as solvent exchange and amine impregnation. Third, solution systems of CA containing solvents and nonsolvents have been extensively reported in the literature for the preparation of CA membranes. The existence of detailed phase diagrams for these systems assists rapid screening and identification of suitable compositions of ternary inks for SBAM.

After the polymer component is identified, it is important to select suitable solvents for the SBAM ink, as solvent volatility is critical for controlling the phase separation speed and corresponding textural properties of the printed monoliths. *N*,*N*-Dimethylacetamide (DMAc) and water were first selected as the solvent and nonsolvent for CA, respectively. The cloud point technique was employed to determine the binodal line of the CA/DMAc/H_2_O ternary system (Figure S1). Although a room-temperature homogeneous ink in the vicinity of the binodal line was successfully identified, it was incapable of rapid phase inversion (i.e., solidification within 1 min after air exposure), which can be attributed to the slow evaporation of the relatively nonvolatile DMAc. Therefore, acetone was selected as a cosolvent, along with DMAc. Because acetone is highly volatile, its evaporation is expected to quickly shift the ink composition away from the solvent pole in the phase diagram and trigger phase inversion after the composition crosses the binodal line. As shown in Figure S1, changing pure DMAc to the mixture of DMAc and acetone (1:1 mass ratio) shifts the position of binodal line to the right and allows for higher nonsolvent (H_2_O) content in the ink. We hypothesize that this leads to greater porosity in the printed structures.^36^

Zeolite 13X was selected as the model adsorbent for incorporation into CA/DMAc/acetone/H_2_O dopes for monolith preparation by SBAM. The CA content was fixed at 15 wt % (excluding zeolite 13X) to achieve good ink fluidity and viscosity; the detailed procedures to prepare the SBAM inks containing zeolite 13X are available in Section 3 of the Supporting Information. The SBAM inks remained homogeneous for at least 1 week after they were prepared. However, these inks did stratify after long-time settling (∼6 months), which is likely because the gradual aggregation of the adsorbent particles accelerates the settling. A customized 3D printer, as illustrated in Figure 1a, was built to deposit polymer filaments containing sorbents. The dope deposition rate was controlled by the N_2_ pressure in the ink cartridge headspace. The print speeds and layer heights were controlled to allow for good adhesion between different layers. CA/13X monoliths were successfully prepared with excellent fidelity compared to the designed structures (Figure 1b). At 50 wt % zeolite 13X loading, the printed monolith had excellent adhesion between different layers of filaments (Figure 1c), and zeolite 13X crystals were randomly distributed in the hierarchical porous CA matrix because of spinodal decomposition (Figure 1d).

However, when zeolite 13X loading increased beyond 60 wt %, the obtained monoliths exhibited dense structures with low porosity (Figure S2a). A possible reason for this could be solvent adsorption in the zeolite 13X particles during ink preparation, which would result in phase inversion of the ink before deposition on the printing platform. To prevent undesired preprinting phase separation of the polymer ink due to addition of zeolite 13X, dry zeolite 13X was presaturated with mixed solvent vapor that was in equilibrium with the mixed solvents used for dope preparation. The vapor-loaded zeolite 13X was subsequently used for printing a monolith (denoted as CA/13X) containing 60 wt % zeolite 13X. This monolith exhibited significantly improved porosity (Figure S2b) compared to monoliths prepared without the presaturation steps. The loading of zeolite 13X can be further increased to 70 wt % without compromising the printing quality of CA/13X monoliths (Figure S2c). Interestingly, no significant differences in BET surface areas and pore volumes were observed between two monolith samples prepared with different zeolite 13X samples (presaturated or not) after taking the different zeolite 13X loadings into consideration (Figure S2d). These results suggest that the presaturation step mainly affects the macroporosity of the monoliths. Measurement of the CO_2_ uptake kinetics of CA/13X monoliths containing 65 wt % zeolite 13X by thermogravimetric analysis (TGA) reveals rapid CO_2_ uptake kinetics (Figure S3a) and a CO_2_ capacity of 2.6 mmol g_monolith_^–1^ (4.0 mmol g_zeolite_^–1^ at 50 kPa and 30 °C), which are comparable to the powder zeolite 13X.^42^ These results suggest that incorporation of zeolite into the monolith structures has negligible adverse effects on the CO_2_ uptake properties of zeolite 13X.

Interestingly, an uneven pore size distribution within the printed structure was observed. For example, the bottoms of the filaments from the upper printed layers are typically more porous than the upper surfaces of the filaments from the lower layers (Figure 1e). Such heterogeneity in pore sizes persists regardless of our efforts to optimize ink compositions. On the other hand, individual filaments extruded by a syringe using the same ink possess evenly distributed pore sizes (Figure S3b). To reconcile these two observations, we speculate that the uneven distribution of pore sizes is related to the 3D printing process. Solvents from newly deposited filaments serve as annealing agents and reduce the surface pore sizes of the filaments from the lower layers.

### 3.2 Fabrication of MIL-101(Cr) Monoliths via SBAM

Monoliths composed of CA and MIL-101(Cr) crystals [denoted as CA/MIL-101(Cr)] were successfully prepared by SBAM using ink formulations, sorbent presaturation, and 3D printing parameters similar to those for preparing CA/13X monoliths. MIL-101(Cr) powder was first synthesized based on a previously reported large-scale production method.^40^ Powder X-ray diffraction (PXRD) patterns reveal the phase purity of the activated MIL-101(Cr) product (Figure S4a). The particle sizes of MIL-101(Cr) crystals were less than 1 μm (Figure S4b), which is beneficial for preparing a well-mixed polymer ink containing MIL-101(Cr) for 3D printing. The N_2_ sorption isotherm at −195.8 °C reveals a BET surface area of 3057 m^2^ g^–1^ and a pore volume of 1.58 cm^3^ g^–1^ of MIL-101(Cr). Because MIL-101(Cr) has a much higher porosity than zeolite 13X (0.34 cm^3^ g^–1^),^43^ the same weight content of MIL-101(Cr) in the ink will consume larger solvent volume fractions compared to 13X, leading to much greater ink viscosity. Therefore, CA concentrations were adjusted to 12.5 wt % (excluding the mass of sorbent) to achieve a printable viscosity for the ink containing MIL-101(Cr). Furthermore, to minimize potential negative effects of solvent annealing on CO_2_ uptake kinetics, the width of the channel walls and monolith walls was set to the width of a single filament, so that each deposited filament will be minimally affected by annealing solvent vapor from peripheral filaments.

**Figure 2:**
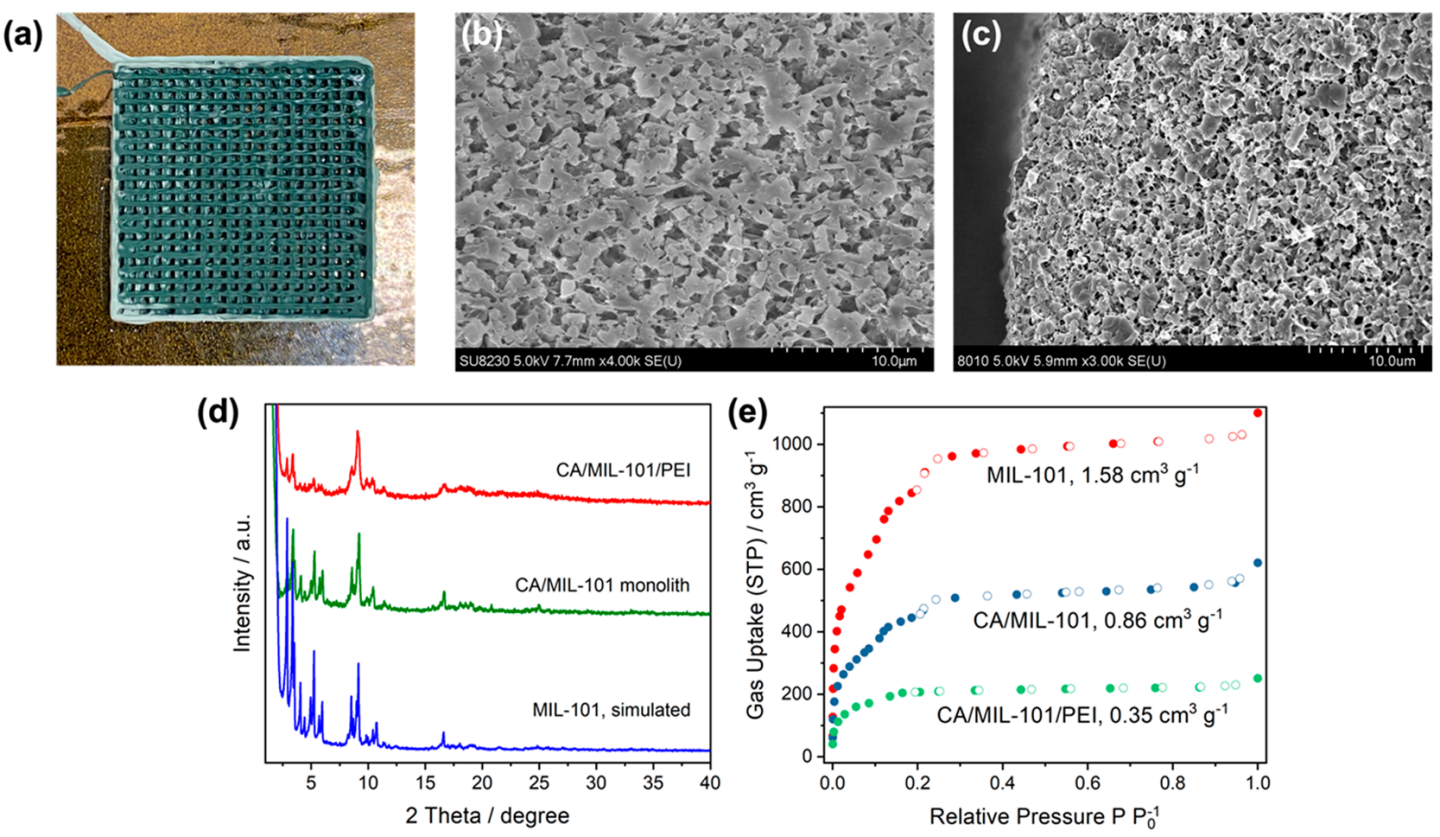
(a) A picture of a CA/MIL-101(Cr) monolith after 3D printing. (b, c) The (b) surface and (c) cross-sectional SEM images of a CA/MIL-101(Cr) monolith show the porosity of the CA network and the homogeneous distribution of nano MIL-101(Cr) crystals in the network. (d) PXRD patterns of MIL-101(Cr) (simulated), CA/MIL-101(Cr) monoliths, and CA/MIL-101/PEI monoliths. (e) N_2_ sorption isotherms of MIL-101, CA/MIL-101(Cr) monoliths, and CA/MIL-101/PEI monoliths at −195.8 °C.

As shown in Figure 2a, the shapes of CA/MIL-101(Cr) monolith channels were well-defined, and the channel number per square inch (CPSI) can be as high as 644 in.^–2^. TGA suggests the MIL-101(Cr) loading in the monolith was 62 wt % (Figure S4c). No dense skin layers were observed on the monolith surface (Figure 2b), and MIL-101(Cr) crystals are evenly distributed in the macroporous CA networks without aggregation (Figure 2c). PXRD patterns of CA/MIL-101(Cr) exhibit characteristic diffraction reflections for MIL-101(Cr), suggesting that MIL-101(Cr) remains crystalline after SBAM and subsequent solvent exchange processes (Figure 2d). The BET surface area of the monolith was calculated to be 1569 m^2^ g^–1^ based on its N_2_ adsorption isotherm at −195.8 °C (Figure 2e), which corresponds well with the surface area of MIL-101(Cr) and 62% loading. Pore size distribution analysis shows that the porosity of MIL-101(Cr) is well preserved (Figure S5a), which, along with monolith macropores, is beneficial for the incorporation of amines and fast diffusion of CO_2_.

### 3.3 Fabrication and CO_2_ Sorption Properties of PEI-Loaded CA/MIL-101(Cr) Monoliths

In our prior work of preparing MIL-101(Cr)-supported amine sorbents for DAC, we observed good agreement between the experimental and theoretical pore volume of PEI-loaded MIL-101(Cr) using the density of branched PEI (*M*_w_ 800).^13^ This finding suggests the effective insertion of PEI-800 inside the pores of MIL-101(Cr) using this procedure. A dual-solvent strategy was employed to maximize the driving force for infusion of poly(ethylenimine) (PEI) into the pores of MIL-101(Cr).^41,44^ Hexane was selected as the nonpolar solvent, and methanol was used as the polar solvent to dilute PEI and load it into the MIL-101(Cr) powder. After PEI infusion, it is crucial to wash the CA/MIL-101(Cr) monoliths with methanol to remove excess PEI that blocks CO_2_ diffusion pathways of CO_2_ to the well-dispersed amine sites in the pores of MIL-101(Cr). TGA combustion experiments show that ∼14 mmol of N g_MOF_^–1^ PEI loading was achieved in a monolith using a typical dual-solvent recipe (32.7 g of hexane, 2.1 g of 33 wt % PEI solution in methanol), which is equivalent to 38.5 wt % PEI in PEI-loaded MIL-101(Cr) immobilized in the monolith. PXRD patterns of the PEI-loaded CA/MIL-101(Cr) monoliths, denoted as CA/MIL-101/PEI-*X*, where *X* indicates a N loading of *X* mmol per gram of MIL-101(Cr), suggest that MIL-101(Cr) particles in the monoliths remain crystalline. The reduced intensity of the diffraction peaks at 2θ values smaller than 7° is attributed to the scattering of unorganized PEI in the pores of MIL-101(Cr) (Figure 2d). Although CA has the potential for hydrolysis in basic and acidic solutions, attenuated total reflection infrared spectroscopy (ATR-IR) (Figure S5b) suggested that the PEI infusing process is a physical process not involving any chemical transformations (e.g., hydrolysis of CA). In addition, SEM shows that the CA framework maintained the same macroporous texture after PEI infusion (Figure S5c). These results together suggest that CA/MIL-101(Cr) monoliths have great stability under PEI loading conditions.

**Figure 3:**
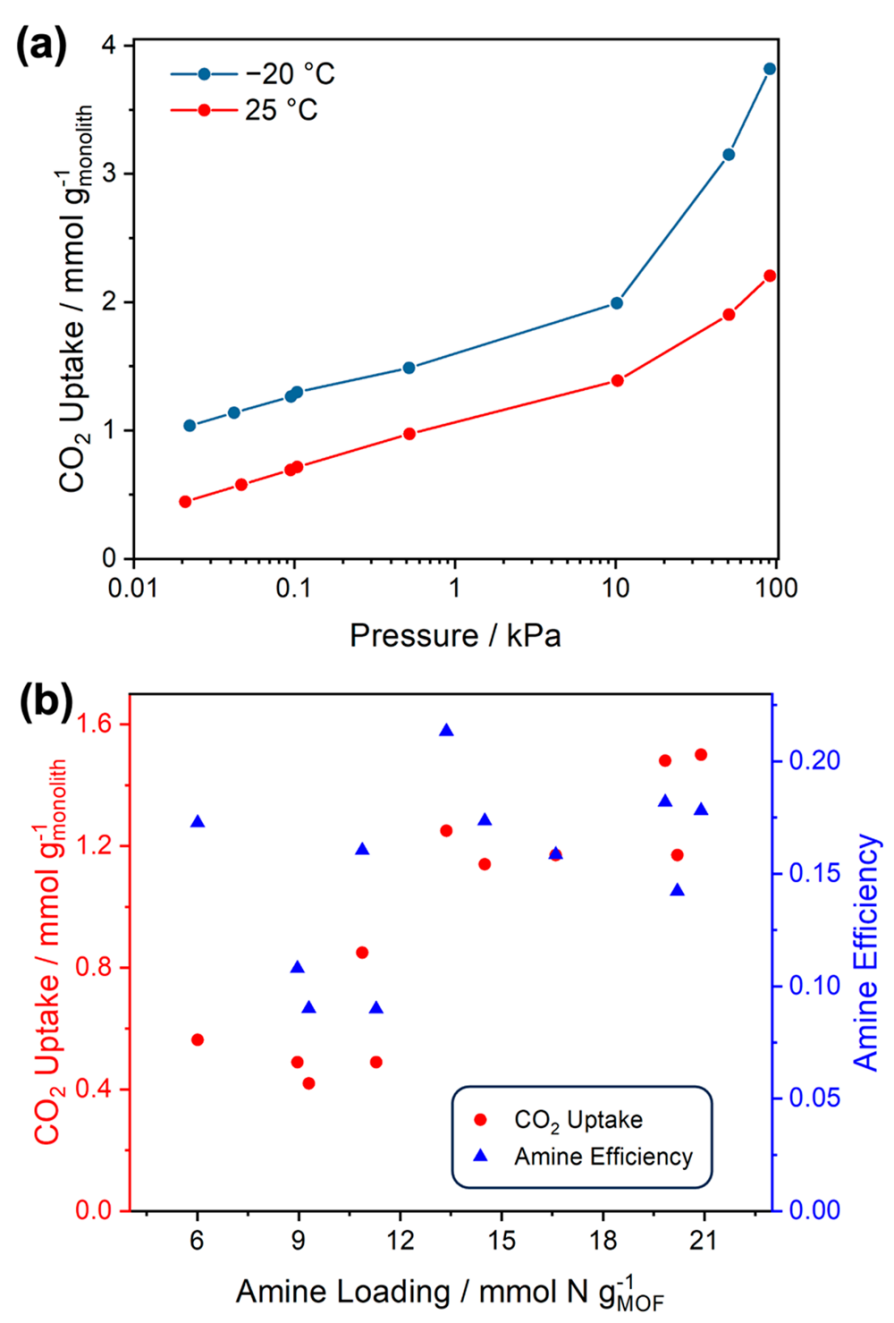
(a) CO_2_ adsorption isotherms of monolith CA/MIL-101/PEI-14.5 at −20 and 25 °C. (b) Comparison of the CO_2_ uptake capacities and amine efficiencies at −20 °C for CA/MIL-101/PEI monoliths with different PEI loadings.

The CO_2_ uptakes of CA/MIL-101/PEI-14.5 at different pressures measured by the volumetric SAP system are shown in Figure 3a. While 25 °C was selected as a representative temperature for ambient conditions, −20 °C was selected as the extreme cold temperature to magnify the temperature effects on DAC performance of amine-loaded MIL-101(Cr) adsorbents and to compare against our prior study.^12^ At 400 ppm, the CO_2_ uptakes in the CA/MIL-101/PEI monolith were 1.1 and 0.57 mmol of g_monolith_^–1^ at −20 and 25 °C, respectively. Assuming that PEI-loaded MIL-101(Cr) provides all the CO_2_ sorption sites and the CA framework only serves as the support, these values correspond to 1.5 and 0.77 mmol g_sorbent_^–1^ at −20 and 25 °C, respectively, which correlates well with our previous work where MIL-101(Cr) powders with comparable PEI loading exhibited similar CO_2_ uptakes under the same testing conditions (Figure S6).^12^ The same PEI loading method was repeated three times to provide consistent CO_2_ uptakes at 400 ppm of CO_2_ and −20 °C, suggesting good reproducibility of this PEI loading method.

As the CO_2_ adsorption heats of CA/MIL-101/PEI monoliths may vary at different temperatures, the commonly used method of measuring several CO_2_ adsorption isotherms at different temperatures with the SAP system and estimating the adsorption heats based on the Clausius–Clapeyron equation may not apply. Therefore, the CO_2_ isosteric heat at 25 °C was directly measured as −80 kJ mol^–1^ by integrating the measured heat flow during the CO_2_ sorption experiment performed on TGA/DSC (Figure S7). This value is between the low adsorption heat of MIL-101(Cr) with low amine content (e.g., 30 wt % TEPA) and the high adsorption heat of MIL-101(Cr) with high amine content (e.g., 50 wt %) found in prior work.^12^

To study how the PEI loading affects the CO_2_ uptake performance, several CA/MIL-101/PEI monoliths with varying amine loadings were prepared by varying the amount of PEI solution used in the PEI infusion step. In general, the amount of incorporated PEI in the monoliths is positively correlated to the amount of PEI used during the PEI infusion step. As shown in Figure 3b, the CO_2_ uptake capacity increased with the amine loading but plateaued when the amine loading was more than 20 mmol N g_MOF_^–1^. The plateau in the CO_2_ uptake might be due to pore blockage at high PEI loadings in MIL-101(Cr). Most monolith samples exhibit amine efficiencies (defined as the moles of sorbed CO_2_ uptake normalized by the moles of amine sites) between 0.15 and 0.20, which are comparable to the amine efficiencies of PEI or TEPA impregnated MIL-101(Cr) powder sorbents.^12^ Some monoliths with relatively low amine loadings (<12 mmol N g_MOF_^–1^) show amine efficiencies that are smaller than 0.12. As these monoliths were treated with small amounts of PEI solution during the PEI infusion process, the small PEI concentration gradients during this step may result in slow PEI diffusion and nonuniform PEI distribution in the monoliths, which eventually lead to poor amine efficiencies. Considering the good reproducibility of the PEI loading experiments, the dual-solvent amine loading method specified in the Experimental Methods section was subsequently used to modify large CA/MIL-101(Cr) monoliths for breakthrough experiments.

**Figure 4:**
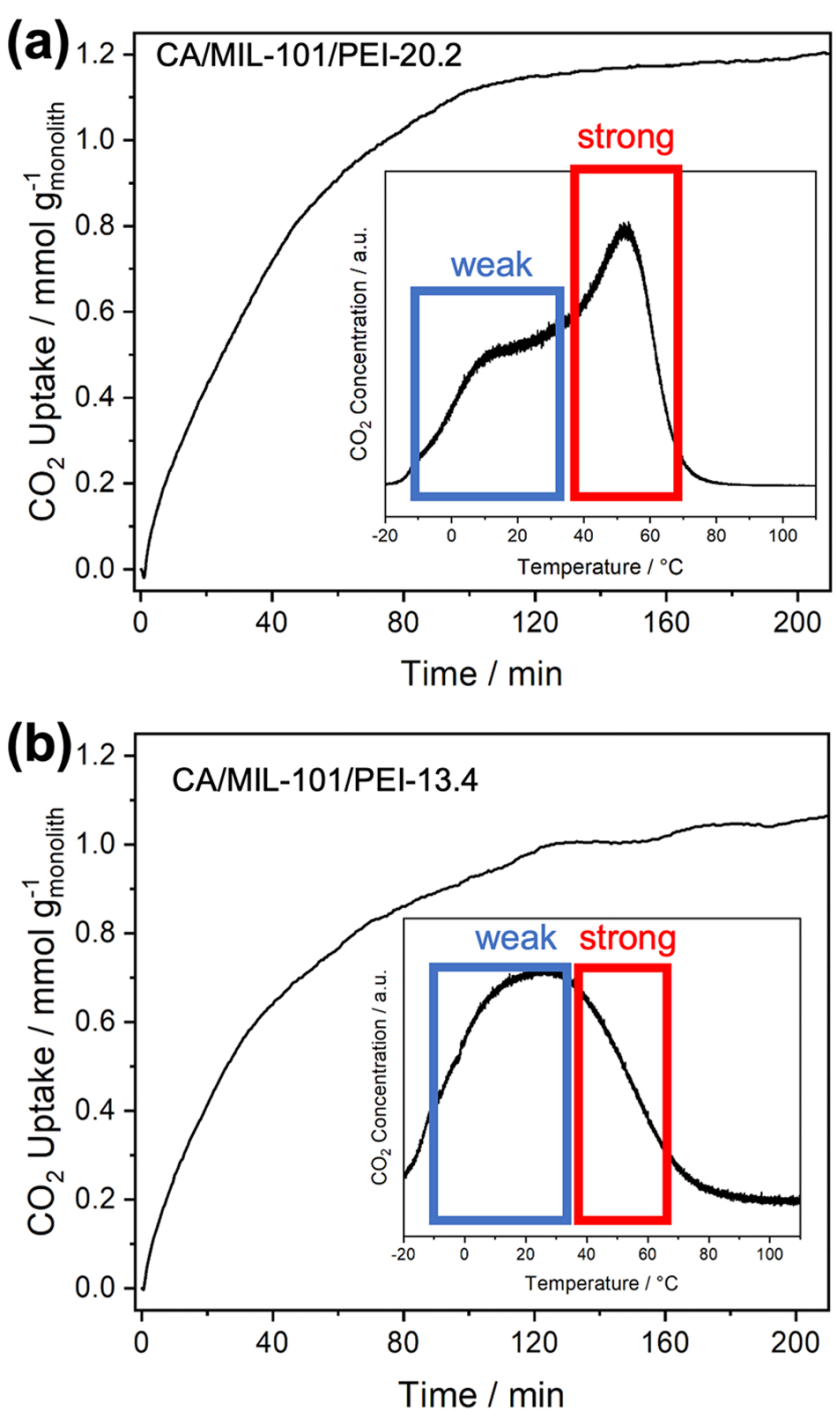
CO_2_ dynamic uptake profiles of (a) CA/MIL-101/PEI-20.2 and (b) CA/MIL-101/PEI-13.4 at −20 °C measured via TGA/DSC. Insets: CO_2_ TPD profiles of the respective CA/MIL-101/PEI monoliths.

Although it is convenient to measure CO_2_ uptakes at different CO_2_ partial pressures and temperatures with the SAP system, it does not provide information on CO_2_ uptake kinetics. Therefore, dynamic uptake profiles (Figure 4) using 400 ppm of CO_2_ at −20 °C were collected by the TGA/DSC setup, which revealed comparable CO_2_ uptake rates for two CA/MIL-101(Cr) monoliths with high (20.2 mmol N g_MOF_^–1^) and low (13.4 mmol N g_MOF_^–1^) PEI loading. Both monoliths reached pseudoequilibrium (*M*/*M*_∞_ = 0.95) in about 2 h, which is similar to the CO_2_ uptake kinetics of PEI-impregnated MIL-101(Cr) in powder form.^12^ This suggests that the CA frameworks have negligible effects on CO_2_ diffusion under the subambient conditions used here, despite the presence of narrow pore sizes at the interfaces between the different layers of filaments. It is worth noting that a higher productivity can be achieved at the process scale with optimized durations of adsorption/desorption steps and higher flow rates.^45^ However, more detailed experiments are needed to further support this supposition. Temperature-programmed desorption (TPD) experiments reveal that interactions of CO_2_ with CA/MIL-101/PEI monoliths are dependent on the PEI loading in the monoliths. For CA/MIL-101/PEI-20.2, a bimodal CO_2_ desorption profile with a peak desorption temperature of 52.3 °C was observed (inset of Figure 4a). In comparison, a unimodal desorption profile with the peak desorption temperature at 26.4 °C was observed for CA/MIL-101/PEI-13.4 (inset of Figure 4b). These results suggest that increased PEI loading provides more strong chemisorption sites for CO_2_ interactions, highlighting the importance of optimizing the PEI loading in these monoliths for a balance of high CO_2_ uptake and facile regeneration. Similar interaction mechanisms dependent on PEI loading have been reported in prior works on PEI-based adsorbents in powder form.^12,46−48^

In addition, the CO_2_ kinetics of CA/MIL-101/PEI pellets of different sizes, pellet-L with large size (2 × 4 × 3 mm^3^) and pellet-S with small size (1 × 0.2 × 5 mm^3^) (Figure S8a), were compared with the monoliths prepared by 3D printing. The detailed procedures to prepare these pellets are available in the Supporting Information. As shown in Figure S8b, the monolith and pellet-S reach a normalized CO_2_ uptake capacity of 0.9 in 160 min, while pellet-L could reach a normalized CO_2_ uptake capacity of only 0.66 in the same amount of time. The much faster CO_2_ sorption kinetics of pellet-S and CA/MIL-101/PEI monoliths suggests the importance of controlling the CO_2_ diffusion lengths in the composites of CA and MIL-101(Cr). Notably, the comparable CO_2_ uptake kinetics in pellet-S and monoliths suggest that the nonuniform pore size distribution (due to repetitive filament deposition and solvent annealing during 3D printing, Figure S8c) does not compromise the CO_2_ diffusion rate or sorption uptake kinetics in the CA/MIL-101/PEI monoliths. This is likely because the average size of the population of these pores might still be too large to change the dominant mass transfer resistance in the monolith, which is the CO_2_ diffusion in the MIL-101(Cr)-supported PEI.

**Figure 5:**
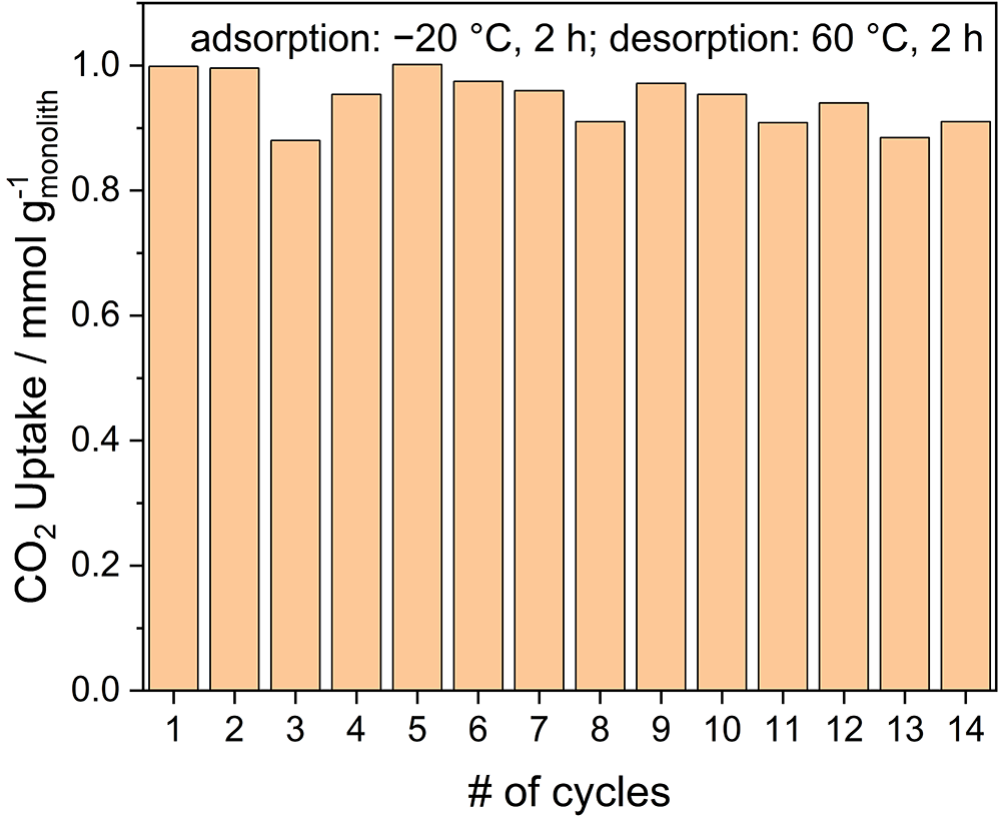
CO_2_ sorption recyclability of CA/MIL-101/PEI-14.5 over 14 adsorption–desorption cycles from TGA/DSC measurements. Adsorption conditions are as follows: gas, 400 ppm of CO_2_/He; flow rate, 90 sccm; *T* = −20 °C; adsorption time, 2 h. Desorption conditions are as follows: gas, He; flow rate, 90 sccm; *T* = 60 °C.

Due to the moderate CO_2_ adsorption heat and CO_2_ affinity of CA/MIL-101/PEI monoliths, less energy is required to desorb equivalent amounts of CO_2_ compared to the case of high heats of adsorption that are found in many DAC sorbents.^12^ A cyclic adsorption–desorption experiment was designed in the TGA/DSC for CA/MIL-101/PEI-14.5 to study its recyclability, with a 2 h CO_2_ adsorption step at −20 °C and a 2 h desorption step at 60 °C. The average working capacity was about 0.95 mmol g_monolith_^–1^ over 14 cycles (Figure 5). The decrease of about 0.15 mmol/g in the third cycle is attributed to an instrumental measurement error as the adsorption and desorption runs were performed continuously and automatically by the TGA/DSC setup. Although the consistent CO_2_ working capacity suggests decent stability of CA/MIL-101/PEI-14.5 over this time frame and a possibility of sorbent regeneration at 60 °C, more detailed process studies will be required to verify the benefit of the low CO_2_ heat of adsorption for DAC at low temperatures.

### 3.4 Mechanical Strength and DAC Performance of CA/MIL-101/PEI Monoliths

After SBAM methods for printing CA/MIL-101(Cr) monoliths were developed, 1.5 cm × 1.5 cm pieces of CA/MIL-101/PEI monoliths were fabricated (Figure S9a) for breakthrough experiments. There are two conceptual ways to prepare such monoliths with large dimensions, namely, a bottom-up method and a “slice and stack” method (Scheme S3). The bottom-up method is relatively straightforward in terms of the printing process. However, the monoliths prepared by this method will deform as their height increases because the recently phase-separated monolith foundation does not have the mechanical strength to support the weight of the growing monolith. For the “slice and stack” method, there are two ways to slice monoliths into small parts. Horizontal slicing has been adopted in the literature; however, as a large number of monolith pieces are required to be stacked into a tall monolith, subsequent alignment of the monolith channels is challenging. In comparison, vertical slicing results in fewer monolith pieces, which is beneficial for obtaining straight channels with little resistance for gas flows. Additionally, it is more efficient to fabricate large monolith pieces for vertical slicing methods than to print many small pieces for horizontal slicing methods from the perspective of large-scale manufacturing via 3D printing. Therefore, the "vertically sliced" method was adopted to prepare CA/MIL-101/PEI monoliths, denoted as monolith-L, for mechanical testing and dynamic column experiments.

The results of compression tests using monolith-L are shown in Figure S9b. Uniaxial force was applied in the *z* direction of the monoliths. Interestingly, the stress gradually increased when the strain was less than 0.15 but increased rapidly for the higher strain region. Consistent results were observed for two monolith samples. Similar mechanical responses for the monoliths have also been reported for 3D-printed zeolite monoliths.^27^ Further inspections of the monolith sample after the mechanical test showed that deformation and delamination mainly occurred in “ridges” of the monolith samples (the channels of the bed when monolith-L are packed together; Figure S9c). In comparison, the “base” of the monolith only underwent slight compression deformation. This is because the ridges have much smaller cross-sectional areas (less than 60% of the base) and hence much greater stress compared to that applied to the base. The base of the monoliths did not break at the maximum loading of the testing device (∼2K N), suggesting decent compressive strength for the monolith-L prepared by SBAM. We envision that further optimization of 3D printing parameters and monolithic structures can improve the adhesion of different 3D-printed layers and enhance the overall mechanical stability.

**Figure 6:**
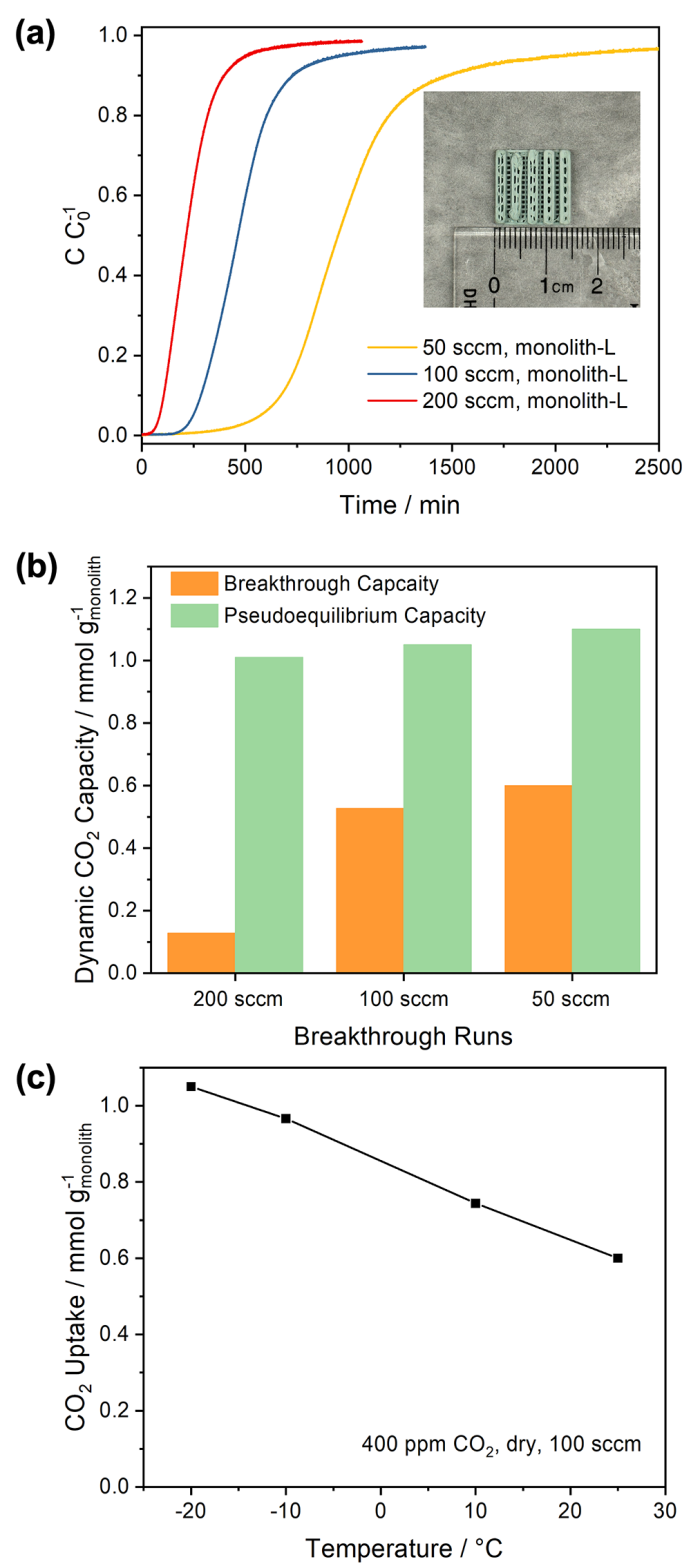
(a) Dynamic breakthrough curves of 400 ppm of CO_2_ at −20 °C of the monolith-L packed bed using different flow rates of feeding gas under dry conditions. (b) Breakthrough (*C*/*C*_0_^–1^ = 0.05) and pseudoequilibrium (*C*/*C*_0_^–1^ = 0.95) CO_2_ capture capacities at −20 °C using different flow rates of feeding gas under dry conditions. (c) Pseudoequilibrium CO_2_ uptake capacities of monolith-L determined by breakthrough experiments at different temperatures.

Monolith-L was packed in a homemade stainless-steel housing (Figure S10a) for dynamic breakthrough experiments using 400 ppm of CO_2_ under dry conditions. As shown in Figure 6a, when the flow rate of the feed gas was 200 sccm at −20 °C, CO_2_ broke through the column almost instantly, possibly due to a CO_2_ bypass through the straight channels of the monoliths. The pseudoequilibrium CO_2_ uptake capacity of these monoliths was 1.05 mmol g_monolith_^–1^, which is consistent with the results of the gravimetric and volumetric CO_2_ uptake measurements. The volumetric CO_2_ uptake of the bed made of monolith-L is calculated to be 0.244 mmol cm^–3^ by using the total bed volume, including the channels, to calculate the apparent monolith density. This value can be further enhanced by increasing the density of 3D-printed filaments in monoliths and reducing the channel size. When the flow rate was reduced to 100 and 50 sccm, the pseudoequilibrium CO_2_ uptake capacities were almost unchanged, as shown in Figure 6b, but the CO_2_ breakthrough uptake capacities of the bed (i.e., the CO_2_ uptake capacity when the normalized CO_2_ concentration reached 0.05) increased to 0.53 and 0.60 mmol g_monolith_^–1^ for gas flow rates of 100 and 50 sccm, respectively. This is likely because the longer residence time in the bed allows more CO_2_ to be captured before breaking through the bed.

Following the dry 400 ppm of CO_2_ breakthrough experiments, a TPD experiment was performed while purging the monoliths with a constant 100 sccm N_2_ flow. A desorption peak temperature of 40 °C was observed (Figure S10b), which is slightly higher than the desorption peak temperatures for testing CA/MIL-101/PEI monoliths with comparable PEI loading (Figure 4b). This difference is possibly due to the external heating of the packed bed in breakthrough experiments which cannot achieve fast and uniform heating of the monoliths as in the case of TPD experiments using much smaller amount of samples performed in the TGA/DSC system. Breakthrough experiments using 400 ppm CO_2_ were also repeated at higher temperatures. As shown in Figure 6c, the CO_2_ uptake capacities of the monoliths decrease as temperature increases, although the monolith was found to still adsorb 0.60 mmol g_monolith_^–1^ CO_2_ at 25 °C under dry conditions, comparable to other shaped DAC sorbents.^18^ These results highlight the potential versatility of CA/MIL-101/PEI monoliths for DAC under different climate environments.

**Figure 7:**
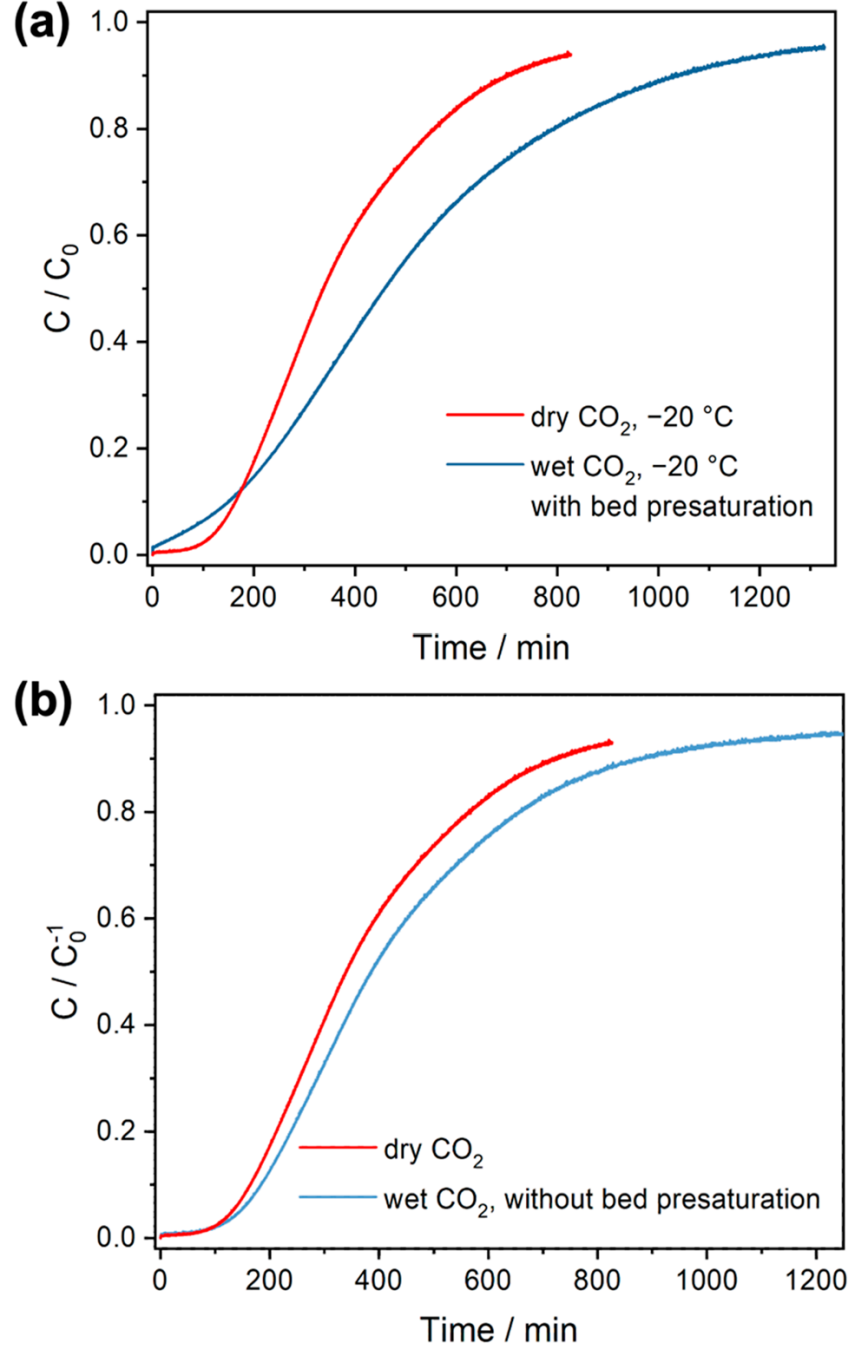
Comparison of 400 ppm of CO_2_ breakthrough curves of the CA/MIL-101/PEI packed bed under dry and wet conditions at −20 °C. (a) The wet CO_2_ breakthrough curve was collected after the CA/MIL-101/PEI monoliths were presaturated by 70% RH at −20 °C before 400 ppm of CO_2_ gas was introduced with 70% RH. (b) The wet CO_2_ breakthrough curve was collected by introducing 70% RH 400 ppm of CO_2_ to the dry CA/MIL-101/PEI −20 °C without presaturating the bed.

The effect of humidity on direct air CO_2_ capture at −20 °C was also explored by presaturating monolith-L with 80% RH moisture in N_2_ followed by the introduction of wet (80% RH) 400 ppm of CO_2_ in N_2_. As the water partial pressure used in the breakthrough experiments (0.8 mbar) was lower than the sublimation vapor pressure of water (0.99 mbar) at −20 °C,^49^ water vapor should not condense in the column, but it might condense/freeze in the pores of the MIL-101(Cr) and perhaps in the pores of the polymer support and affect the kinetics of CO_2_ adsorption. As shown in Figure S11, moisture broke through the column instantly with a gradually increasing moisture signal at the column exit throughout the presaturation experiment, which suggests sluggish moisture uptake kinetics of the monoliths at −20 °C. The water uptake calculated from the breakthrough experiment is about 21.0 mmol g_monolith_^–1^, which is slightly higher than the water uptake measured gravimetrically at 78% RH at 25 °C (Figure S12). Interestingly, an instantaneous CO_2_ breakthrough from the column was also observed in the wet CO_2_ breakthrough experiment. In contrast to the CO_2_ breakthrough curves under dry conditions, the significantly broader breakthrough curve of wet CO_2_ suggests a noticeable decline of CO_2_ uptake kinetics (Figure 7a), which is possibly due to the additional mass transfer resistance originating from preadsorbed water molecules around amine sites or perhaps in the mesopores/macropores of the CA polymer matrix. Despite the decreased CO_2_ uptake kinetics, the CO_2_ uptake capacity increased by about 36% from 1.05 mmol g_monolith_^–1^ under dry conditions to 1.43 mmol g_monolith_^–1^ in the wet monoliths. This enhanced CO_2_ uptake under humid conditions is attributed to the improved chain mobility of PEI molecules due to the plasticizing effects of water^12^ and/or enabling CO_2_ sorption as bicarbonate.^14,50,51^

While some DAC processes will likely operate with constantly hydrated sorbents (e.g., DAC in humid regions using direct contact steam-stripping desorption), in some DAC processes, humid air will be fed into a dry or partially dry DAC bed. As it takes much longer for the bed to saturate with water due to the slow water sorption kinetics, the water uptake in the bed at the end of the CO_2_ adsorption step should be much lower than the maximum uptake capacity in these latter types of DAC processes. To mimic this specific scenario, wet 400 ppm of CO_2_ with 80% RH moisture was directly introduced to the dry bed of CA/MIL-101/PEI monoliths without presaturation of the bed. Interestingly, the mean residence time of CO_2_ became longer (Figure 7b), and the pseudoequilibrium of the CO_2_ uptake of monolith-L was increased by 22% compared to the dry condition. Meanwhile, the coadsorbed water capacity was 7.0 mmol g_monolith_^–1^, which is about 1/3 of the water uptake capacity (21.0 mmol g_monolith_^–1^) determined by the water presaturation breakthrough curve (Figure S11). This observation suggests that optimizing the duration of the adsorption step could potentially benefit the overall CO_2_ working capacity of the process without paying the substantial energy penalty to remove excessive coadsorbed water molecules.

## 4 Conclusions

In summary, this work demonstrates the utilization of SBAM to fabricate polymer/sorbent composite monoliths with hierarchical porosity and high sorbent content (up to 70 wt %). This work suggests that SBAM can be a useful tool to fabricate contactors of sorbent particles with various pore volumes and chemical compositions, such that the resulting contactors could be potentially used for different chemical separation problems. As a demonstration, CA monoliths containing zeolite 13X or MIL-101(Cr) were successfully fabricated and characterized. PEI was successfully loaded into the CA/MIL-101(Cr) monoliths to fabricate DAC contactors that were then evaluated under both ambient and cold temperature operating conditions. Integration of PEI-loaded MIL-101(Cr) sorbents into the macroporous CA network does not compromise their DAC properties when compared to the powder form, and an average CO_2_ working capacity of 0.94 mmol g_monolith_^–1^ was observed when CO_2_ was adsorbed at −20 °C and desorbed at 60 °C. Under dry conditions, dynamic breakthrough experiments at −20 °C showed a pseudoequilibrium CO_2_ uptake capacity of 1.05 mmol g_monolith_^–1^, which is consistent with single-component CO_2_ sorption results. Presaturating the bed with 70% RH moisture boosted the pseudoequilibrium CO_2_ uptake to 1.43 mmol g_monolith_^–1^ at −20 °C, which is attributed to the plasticizing effects of moisture or the formation of bicarbonate species. Combined with 400 ppm of CO_2_ breakthrough experiments performed at temperatures below 15 °C, this study suggests the potential applicability of CA/MIL-101/monoliths in DAC under subambient conditions.

Key limitations of this work include the moderate MIL-101(Cr) loading (< 70 wt %) in the monoliths and the relatively simple monolith structures achieved so far. It would be desirable to harness the printing versatility of SBAM to fabricate monoliths with more complex geometries and compare their performance. Preliminary results show that SBAM could be employed to fabricate monoliths with gyroid channels (monolith-G, Figure S13a) using gyroid as the infill pattern and 25% as the infill density in Cura. In comparison to monolith-L, the bed made of monolith-G showed breakthrough curves of 400 ppm of CO_2_ under dry conditions at −20 °C with similar pseudoequilibrium CO_2_ uptake capacities and mass transfer performance (Figure S13b), suggesting that SBAM has versatility in preparing monoliths with complex structures without compromising the CO_2_ uptake capacities and kinetics. It should be noted that the linear gas velocities used in these preliminary comparison breakthrough experiments are lower than 1.5 cm s^–1^, which are far from the practical air velocities desirable for DAC processes. Higher gas flow rates in the breakthrough experiments and other forms of gyroid channels might be required to distinguish the differences between the mass/heat transfer dynamics of monoliths with gyroid structures and simple straight-channel structures. In addition, although SBAM and other 3D printing techniques exhibit prospects of affording adsorbent monoliths with complex, unconventional structures, it is challenging to manufacture these monoliths at the scales and speeds needed for DAC. Further study in the mechanical engineering (e.g., 3D printer customizations) and materials engineering (e.g., ink formulation engineering) are suggested for making 3D printing viable for practical manufacturing of DAC contactors. Finally, although a milder regeneration temperature can be used for low-temperature DAC sorbents based on the consistent CO_2_ working capacity obtained in the cyclic experiments, detailed process studies are required to identify optimized desorption conditions for low-temperature DAC sorbents and compare the energy consumption of DAC processes deployed under different climate conditions.

## Supporting Information Available

Schematic illustrations for experimental setups, phase diagram of SBAM ink, characterization data (SEM, TGA, XRD, ATR-IR, pore size distribution), CO_2_ adsorption behavior (TGA/DSC data, adsorption isotherms, CO_2_-TPD data, and breakthrough experiments) of the CA/MIL-101/PEI monoliths (PDF)
