# Supplementary material for: Cold Temperature Direct Air CO2 Capture with Amine-Loaded Metal–Organic Framework Monoliths
Source: ACS Appl Mater Interfaces. 2023 Dec 18;16(1):1404–15. doi: 10.1021/acsami.3c13528 (PMC10788822; doi:10.1021/acsami.3c13528)
Supplement: Supplementary file 1 — am3c13528_si_001.pdf [file am3c13528_si_001.pdf]

Supporting Information

Cold Temperature Direct Air CO_2 Capture with Amine-Loaded Metal-Organic Framework Monoliths

Yuxiang Wang, Guanhe Rim, MinGyu Song, Hannah E. Holmes, Christopher W. Jones, Ryan P. Lively**

School of Chemical and Biomolecular Engineering, Georgia Institute of Technology, 311 Ferst Dr., Atlanta, GA 30332 USA

Email: cjones@chbe.gatech.edu; ryan.lively@chbe.gatech.edu

Table of Contents

1. **Equipment and characterizations** ········ S3

2. **Determination of phase diagrams** ········ S3

3. **Preparation of inks of solution-based additive manufacturing (SBAM) for printing zeolite 13X monoliths** ········ S4

4. **Preparation of CA/MIL-101(Cr) pellets** ········ S4

5. **Breakthrough experiments** ········ S5

Scheme S1. A schematic illustration of the experimental setup to infuse PEI into CA/MIL-101(Cr) monoliths via the dual-solvent method ········ S8

Scheme S2. A schematic illustration of the sub-ambient DAC fixed bed system for breakthrough experiments. ········ S8

Scheme S3. Schematic illustrations of the concepts to assemble sorbent monoliths. ········ S9

Figure S1. The ternary phase diagrams of CA, solvent(s), and nonsolvent. The dashed blue line represents the estimated binodal line of the ternary system where DMAc is the only solvent, while the solid blue line represents the possible binodal line of the ternary system where acetone is used as a cosolvent with DMAc. ········ S10

Figure S2. (a) SEM image of the dense structure of CA/13X monoliths with 60 wt% zeolite 13X content without presaturating zeolite 13X with solvent vapor before preparation of the SBAM inks. (b) Significantly improved porosity was observed in CA/13X monoliths when zeolite 13X was pre-saturated with solvent vapor. **(c)** TGA trace of CA/13X monoliths with 70 wt% loading of zeolite 13X. Insets show the pictures of these CA/13X monoliths. (d) N_2 sorption isotherms at 77 K of CA/13X monoliths prepared with and without sorbent presaturation. The filled and hollow

symbols stand for the adsorption and desorption branches of the isotherms, respectively. The N_2 sorption isotherms of zeolite 13X from literature are plotted for comparison.[1] ·················· S11

Figure S3. (a) Dynamic CO_2 uptake profiles of zeolite 13X powder and CA/13X monoliths with different channel widths measured by TGA. (b) SEM image of a CA/13X filament prepared by syringe extrusion ··· S12

Figure S4. (a) Experimental and simulated PXRD patterns of MIL-101(Cr). (b) The SEM image of MIL-101(Cr) crystals. (c) TGA traces of MIL-101(Cr) and CA/MIL-101(Cr) monoliths. ·· S13

Figure S5. (a) Pore size distribution of MIL-101(Cr), CA/MIL-101(Cr) monoliths, and CA/MIL-101/PEI. (b) FTIR spectra of CA, MIL-101(Cr), CA/MIL-101(Cr) monoliths, and CA/MIL-101/PEI. The green, yellow, and cyan regions highlight the wavenumber positions of C-H from the methyl groups of CA, C=O from the acetyl groups of CA, and C-O from the carboxylates of MIL-101(Cr). (c) SEM image of CA/MIL-101/PEI monolith after PEI infusion.················ S14

Figure S6. Comparison of CO_2 uptake capacities of CA/MIL-101/PEI-14.5 (normalized to the MIL-101/PEI content in the monolith) and PEI-loaded MIL-101(Cr) powder at −20 °C. ······ S15

Figure S7. The CO_2 dynamic uptake profile and DSC trace of CA/MIL-101/PEI-14.5 at 25 °C. ··· S15

Figure S8. (a) Images of CA/MIL-101/PEI pellets. (b) Comparison of CO_2 dynamic uptake profiles of CA/MIL-101/PEI pellets and monoliths at −20 °C. (c) SEM image of the interface between the bottom and top 3D printed-layers of CA/MIL-101/PEI monolith. ·················· S16

Figure S9. (a) A scheme showing the staggering of CA/MIL-101/PEI monoliths (monolith-L) for breakthrough experiments. (b) Mechanical responses of monolith-L as a function of compressive strains. The stress is calculated by the load divided by the cross-section area of the base of the

monoliths. (c) Pictures of monolith-L before and after compression tests from the side and top perspectives. ········ S17

Figure S10. (a) The cross-section and top view of the home-made stainless-steel housing for CA/MIL-101/PEI monoliths. (b) CO_2 temperature-programmed desorption profile of CA/MIL-101/PEI monoliths after a breakthrough experiment of dry 400 ppm CO_2 at −20 °C. ········ S18

Figure S11. The water breakthrough curve during the pre-saturation step of CA/MIL-101/PEI monoliths at −20 °C using a flow rate of 200 sccm. ········ S19

Figure S12. Water uptakes in CA/MIL-101/PEI-13.3 at 25 °C measured gravimetrically. ···· S19

Figure S13. (a) Top views of monolith-L and monolith-G. (b) Breakthrough curves of dry 400 ppm CO_2 of fixed beds using monolith-L and monolith-G at −20 °C. ········ S20

Figure S14. CO_2 uptakes in MIL-101(Cr)/PEI (10 mmol N g_{MOF}^{-1}) at different temperatures. S21

Table 1. Summary of CO_2 uptakes and half-adsorption time of recent DAC sorbents ········ S22

References ········ S23

1. Equipment and characterizations

Scanning electron microscopy (SEM) images were obtained with a Hitachi SU8010 or SU8230. Before imaging, samples were sputtered with a Hummer 6 Gold sputterer for 40 s. PXRD patterns of samples were collected from a X'Pert Pro PANalytical (Malvern Panalytical) diffractometer at 40 kV and 40 mA (Cu Kα source, λ = 1.541 nm). The patterns were collected with a step size of 0.017° 2θ and scan time of 19.68 s/step over 2θ range of 1.5-40°. N_2 physisorption experiments at -195.8 °C were measured with a Belsorp MAX (MicrotracBEL, Japan). Prior to isotherm estimation, the samples were degassed under vacuum below 10^{-2} kPa for 12 h at 150 °C. Isotherms were obtained, and pore size distributions were estimated from the isotherms with 2D-NLDFT (two-dimensional non-local density functional theory) for carbon samples with slit pores (MicroActive software package, Micromeritics). Compression tests were performed on a Mark-10 ESM303 mechanical tester under 0.2 mm min^{-1} compression. Water uptakes at 25 ℃ were measured gravimetrically on a TA Instruments TGA 550. Humidity of the purging gas for the water uptake measurement was set by adjusting the flow rates of a stream of dry N_2 and a stream of humid N_2 whose humidity is regulated by a LICOR portable dew point generator LI-650.

2. Determination of multi-component phase diagrams for cellulose acetate (CA) in the mixture of acetone, DMAc, and H_2O.

The ternary phase diagram for CA/acetone/DMAc/H_2O was determined by the cloud-point technique. CA solutions with different compositions were prepared by mixing DMAc, acetone, H_2O, and CA sequentially in a glass vial. After rapid dispersion aided by a vortex mixer, these CA solutions were then transferred onto a roller and heated by heating lamps to around 50 °C. After slow rotation mixing for 2 d, the solutions were cooled down to 25 °C. After that, the clarity and

fluid flow properties were examined by eye. A flowing clear liquid indicates a homogenous phase while any other appearance indicates a two-phase system. The binodal curve (the boundary between the homogenous region and two-phase region) was located by comparing adjacent dopes that exhibited a homogenous composition and an inhomogeneous composition.

3. Preparation of inks of solution-based additive manufacturing (SBAM) for printing zeolite 13X monoliths

A typical procedure to prepare the SBAM ink for obtaining zeolite 13X monoliths with 75 wt% sorbent loading is shown as follows. First, zeolite 13X powder was activated at 120 °C in vacuum overnight to remove residual solvents in the pores. After activation, the powder was sealed in a jar containing a mixture of DMAc, acetone, and H_2O with the same compositions as the SBAM ink for 7 days to saturate the pores with solvent vapor. The vapor loading in zeolite 13X was determined by TGA. Second, a stock solution of acetone (5.0 g), DMAc (5.0 g), and H_2O (2.7 g) was prepared, and 0.3 g CA was dissolved in 1.7 g stock solution to prepare a prime dope. Third, vapor-saturated zeolite 13X (3.0 g, contains 25 wt% vapor of the mixed solvent) was dispersed in 6.8 g stock acetone/DMAc/H_2O solution by sonication in water bath for 1.5 h before combining this dispersion dope with the prime dope. More vapor-saturated zeolite 13X (3.15 g) was added to the mixture under stirring, and the mixture was further sonicated in water bath for 1.5 h and homogenized by the Branson 450 Digital Sonifier with 20% amplitude for 2 min 20s (20 s pulse with 20 s interval). Fourth, the dispersion dope was combined with the prime dope, and remaining CA (1.2 g) was added to the mixture under stirring. The vial containing the final mixture was subsequently put on a roller under an infrared lamp for at least 3 days to homogenize the ink before SBAM.

4. Preparation of CA/MIL-101(Cr) pellets

CA/MIL-101(Cr) pellets, pellet-S and pellet-L, were prepared by extruding the ink solution for SBAM from the 3D printer nozzle or a syringe. In the case of pellet-S, the SBAM ink was extruded out from the nozzle by N_2 and deposited onto the 3D printer platform, during which N_2 pressure was maintained at above 138 kPa to guarantee the formation of continuous CA/MIL-101(Cr) lines. The lines were subsequently soaked in H_2O for 3 days (water refreshed every day) to achieve complete phase inversion, after which the lines were soaked in methanol and hexane for solvent exchange. The lines were dried in vacuum oven at 100 °C under vacuum before PEI loading using the dual-solvent method. The PEI-loaded CA/MIL-101(Cr) lines were chopped into small pellets with an average dimension of 1 × 0.2 × 5 mm. These small pellets were denoted as pellet-S. Similarly, the SBAM ink was extruded out from a syringe with a diameter of 2 mm and deposited on an alumina foil. The extrudates were soaked in H_2O for complete phase inversion followed with solvent exchange in methanol and hexane. The extrudates were further chopped into small pieces with an average dimension of 2 × 4 × 3 mm, dried, and loaded with PEI using the dual-solvent method. The PEI-loaded chopped extrudates were denoted as pellet-L.

5. Breakthrough experiments

A schematic illustration of the custom-built fixed bed system for breakthrough experiments is shown in **Scheme S2**. Before breakthrough experiments, the bed was activated under 200 sccm N_2 purging at 90 °C for 12 h. The bed was heated by a heating tape, and the temperature was controlled by the Platinum Series universal benchtop PID controller from Omega Engineering. After activation, the bed was immersed in a bath of ethylene glycol and water for at least 30 min before breakthrough experiments at set temperatures regulated by Julabo CD-600F. For breakthrough

experiments under dry conditions, a stream of 400 ppm CO_2 balanced by N_2 was introduced into the bed, and the concentrations of CO_2 and H_2O at the outlet of the bed were recorded by LICOR LI-850 CO_2/H_2O gas analyzer. For breakthrough experiments under wet conditions, the relative humidity of the feed gas was regulated by a dew point generator LICOR LI-650 dew point generator.

The gas (CO_2 or moisture) uptake capacities, q_i, were calculated based on the breakthrough curves using the following equation.

$$q_i = \frac{p\dot{V}y_i}{mRT}\int\left(1-\frac{C_{t,i}}{C_{0,i}}\right)dt \qquad \text{Equation S1}$$

In this equation, p is the bed pressure in Pa, $\dot{V}$ is the volumetric follow rate in m^3 s^{-1}, y_i is the composition of gas species i in the feeding gas stream, R is the gas constant, T is temperature in K, m is the sorbent mass in g, $C_{t,i}$ is the concentration of gas species i recorded by LICOR-850 at the moment t, and $C_{0,i}$ is the concentration of gas species i in the feed gas stream.

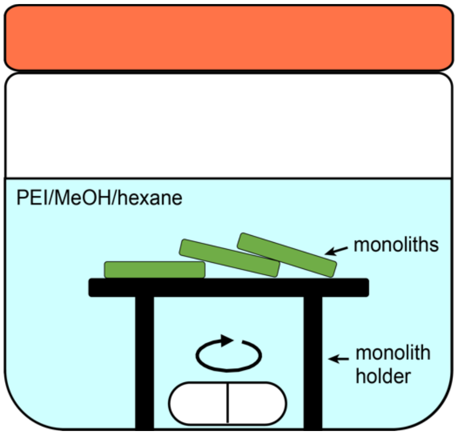

Scheme S1. A schematic illustration of the experimental setup to infuse PEI into CA/MIL-101(Cr) monoliths via the dual-solvent method.

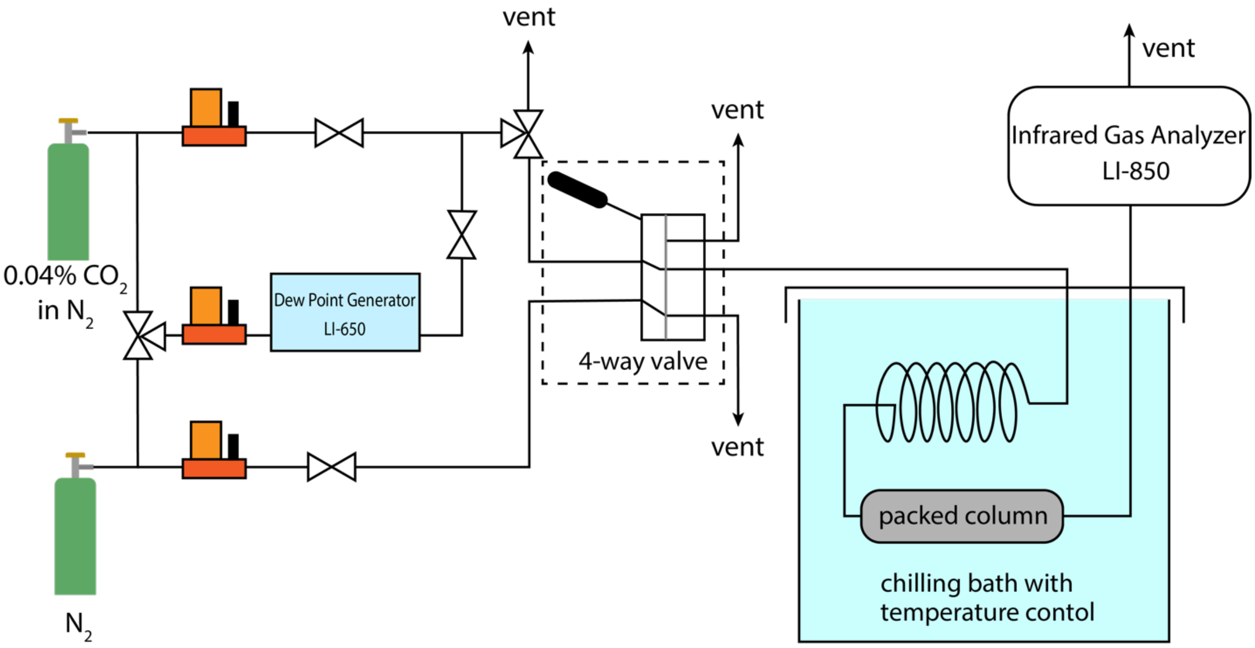

Scheme S2. A schematic illustration of the sub-ambient DAC fixed bed system for breakthrough experiments.

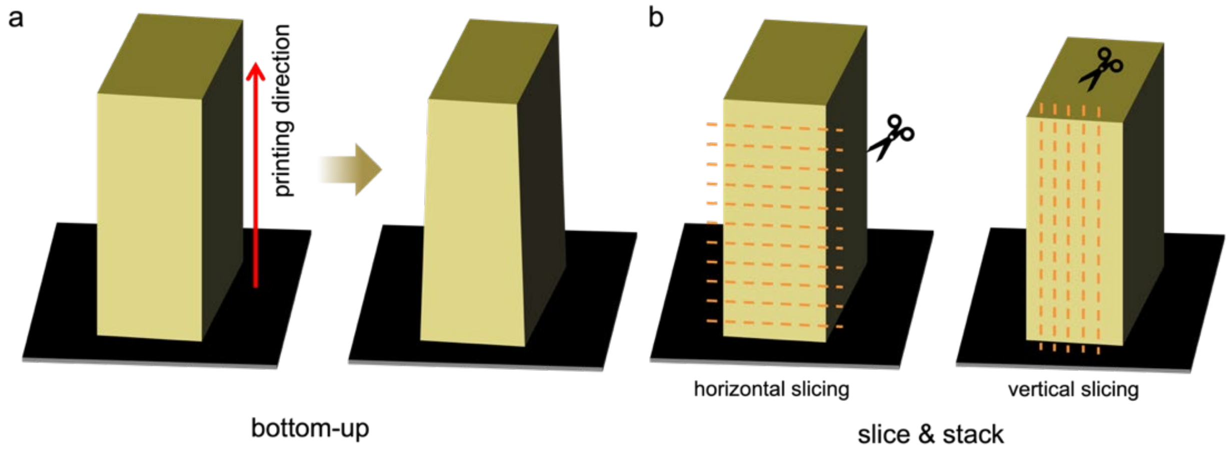

Scheme S3. Schematic illustrations of the concepts to assemble sorbent monoliths.

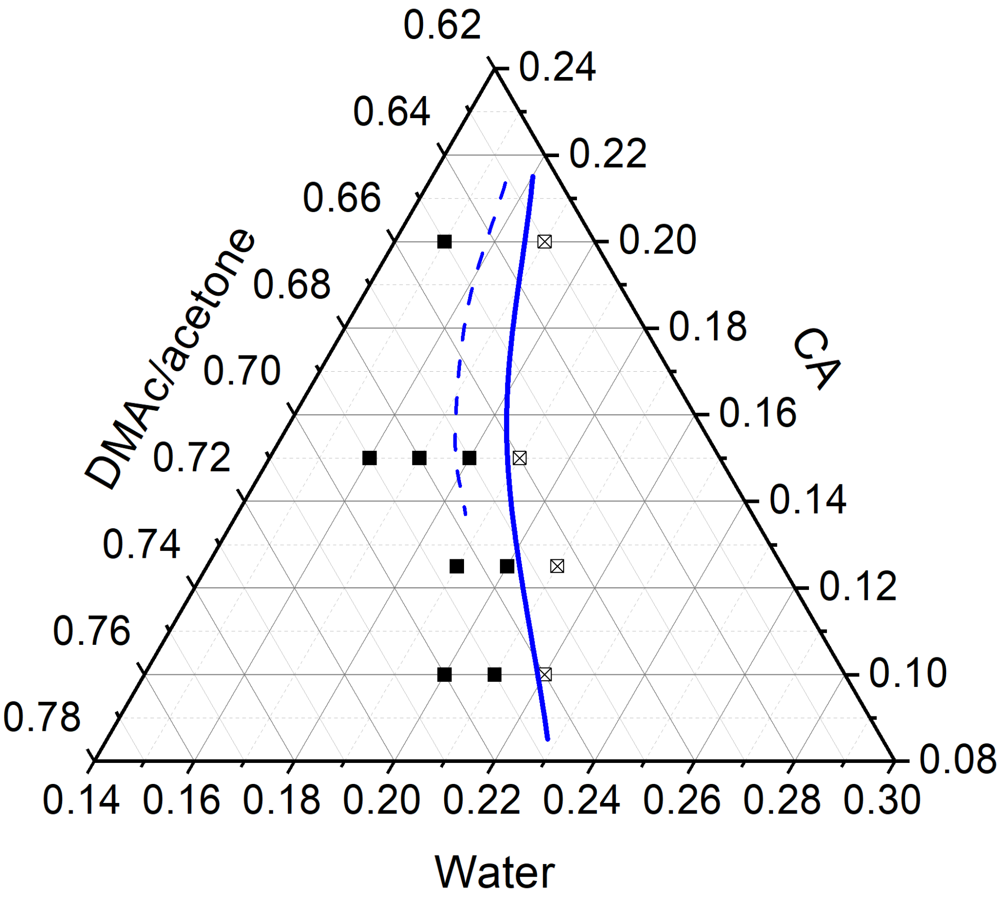

Figure S1. The ternary phase diagrams of CA, solvent(s), and nonsolvent. The dashed blue line represents the estimated binodal line of the ternary system where DMAc is the only solvent, while the solid blue line represents the estimated binodal line of the ternary system where acetone is used as a cosolvent with DMAc in a 1:1 weight ratio.

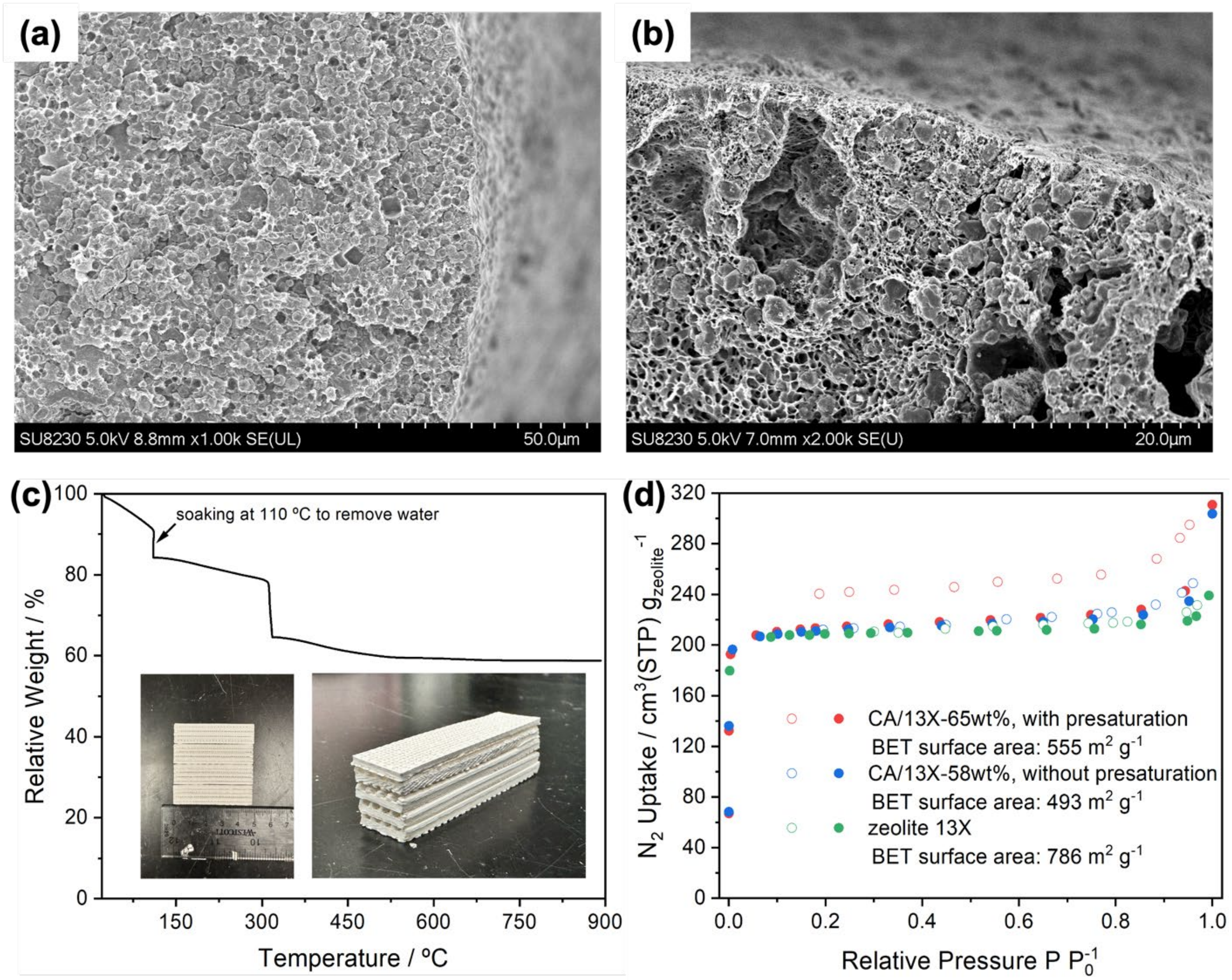

Figure S2. (a) The SEM image of the dense structure of CA/13X monoliths with 60 wt% zeolite 13X content without presaturating zeolite 13X with solvent vapor before preparation of the SBAM inks. (b) Significantly improved porosity was observed in CA/13X monoliths when zeolite 13X was pre-saturated with solvent vapor. (c) TGA trace of CA/13X monoliths with 70 wt% loading of zeolite 13X. Insets show the pictures of these CA/13X monoliths. (d) N_2 sorption isotherms at 77 K of CA/13X monoliths prepared with and without sorbent presaturation. The filled and hollow symbols stand for the adsorption and desorption branches of the isotherms, respectively. The N_2 sorption isotherms of zeolite 13X from literature are plotted for comparison.[1]

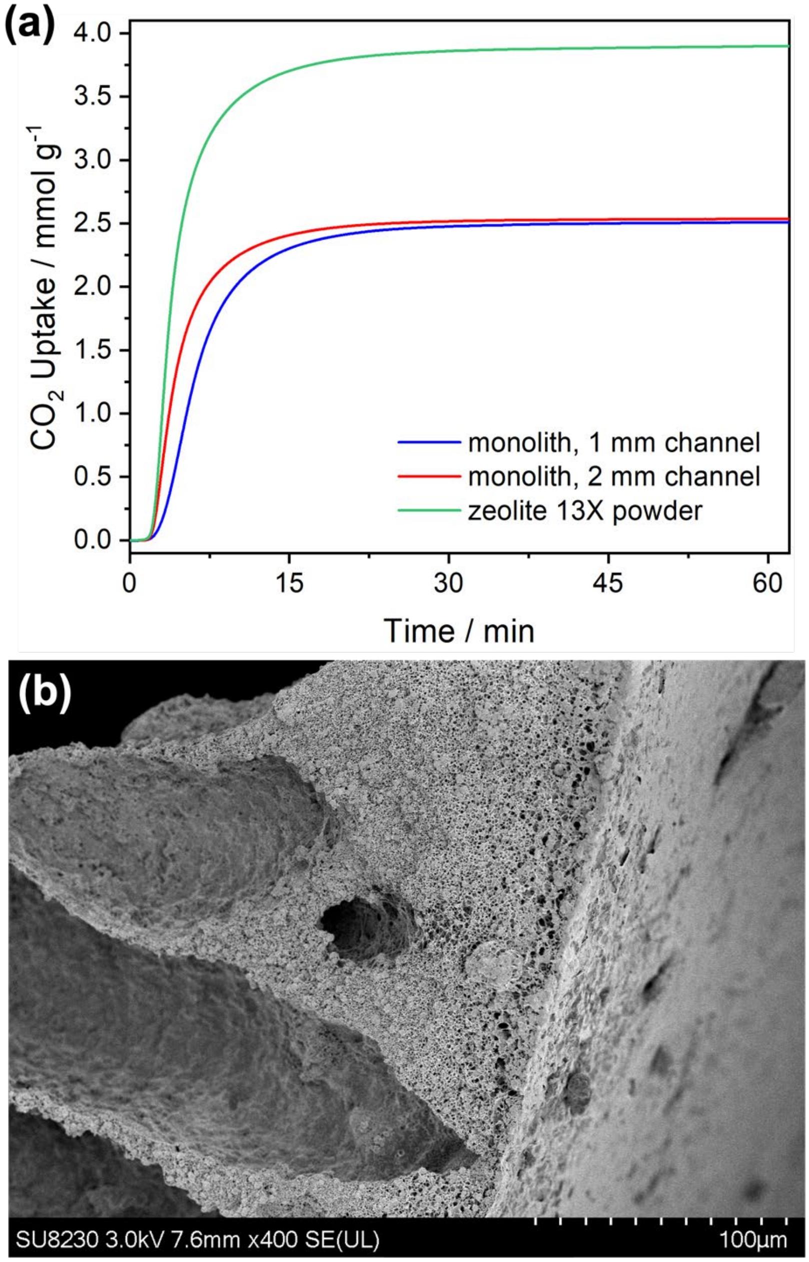

Figure S3. (a) Dynamic CO_2 uptake profiles of zeolite 13X powder and CA/13X monoliths with different channel widths measured by TGA. (b) SEM image of a CA/13X filament prepared by syringe extrusion.

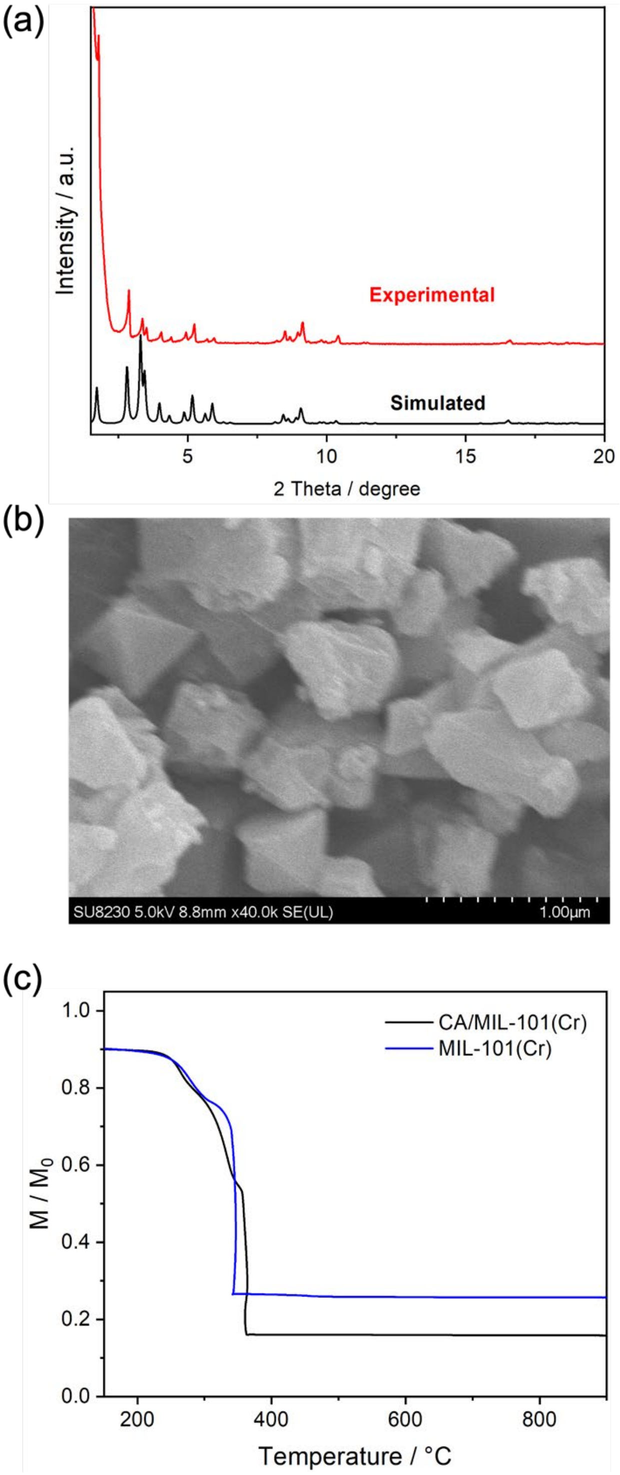

Figure S4. (a) Experimental and simulated PXRD patterns of MIL-101(Cr). (b) The SEM image of MIL-101(Cr) crystals. (c) TGA traces of MIL-101(Cr) and CA/MIL-101(Cr) monoliths.

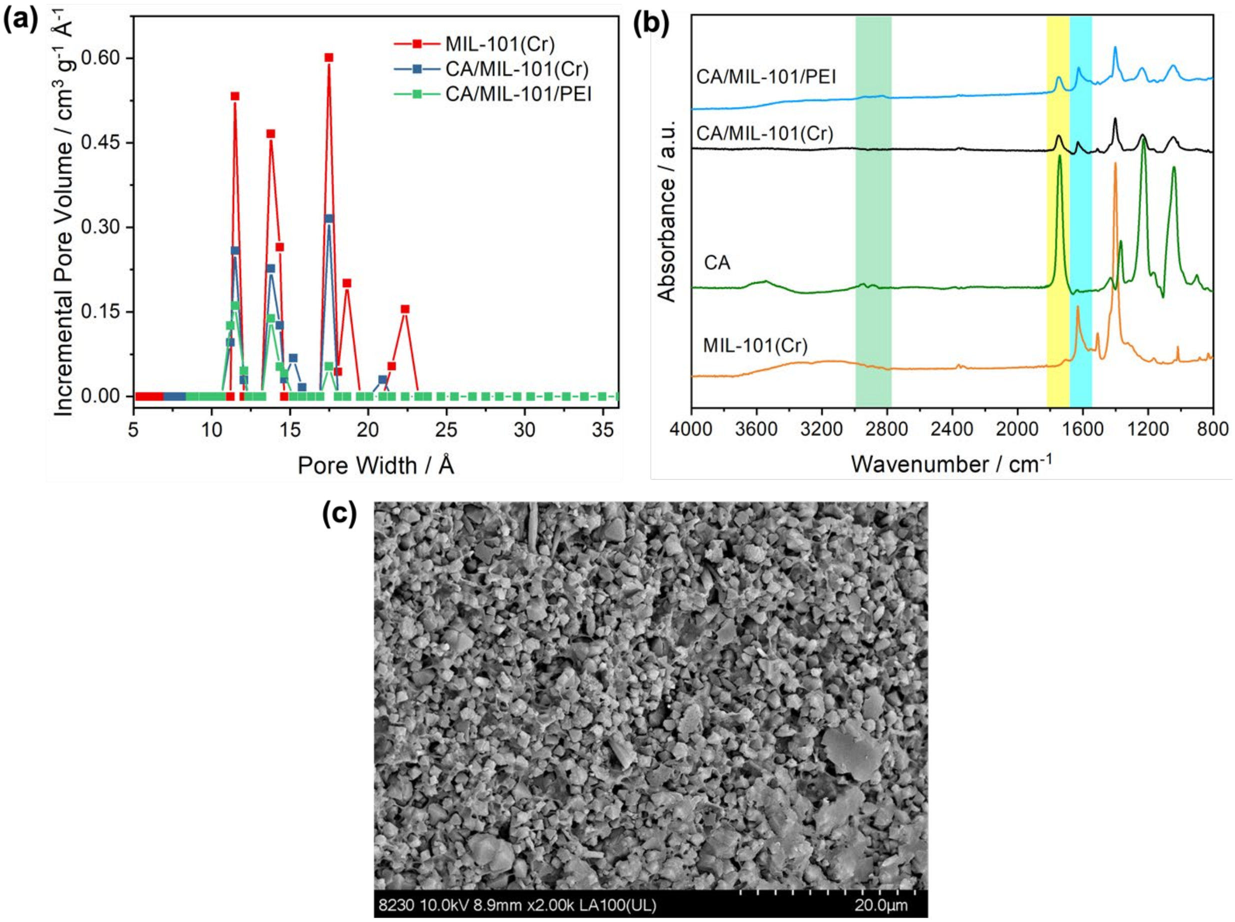

Figure S5. (a) Pore size distribution of MIL-101(Cr), CA/MIL-101(Cr) monoliths, and CA/MIL-101/PEI. (b) FTIR spectra of CA, MIL-101(Cr), CA/MIL-101(Cr) monoliths, and CA/MIL-101/PEI. The green, yellow, and cyan regions highlight the wavenumber positions of C-H from the methyl groups of CA, C=O from the acetyl groups of CA, and C-O from the carboxylates of MIL-101(Cr). (c) SEM image of CA/MIL-101/PEI monolith after PEI infusion.

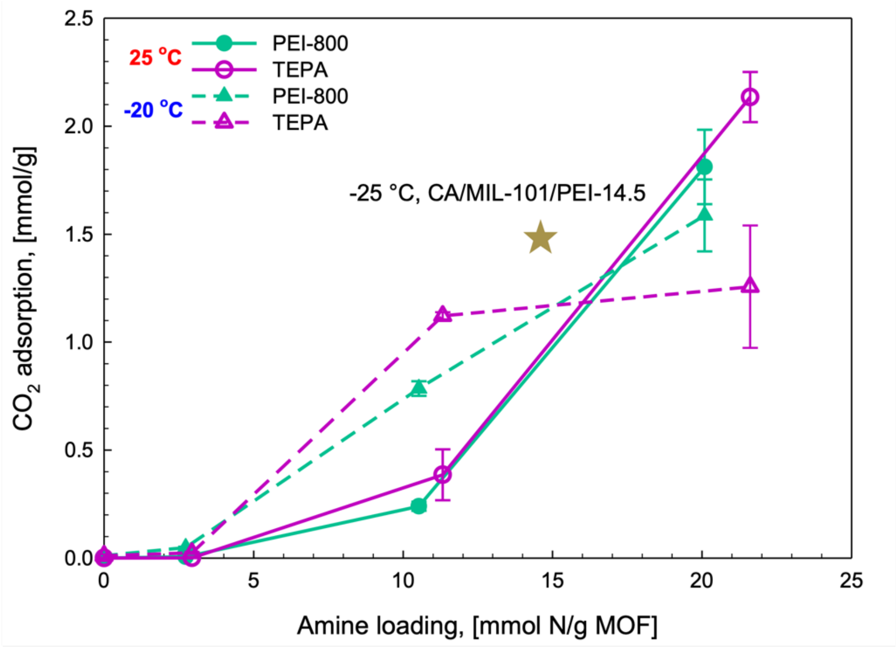

Figure S6. Comparison of CO_2 uptake capacities of CA/MIL-101/PEI-14.5 (normalized to the MIL-101/PEI content in the monolith) and previously-published PEI-loaded MIL-101(Cr) powder at −20 °C.[2]

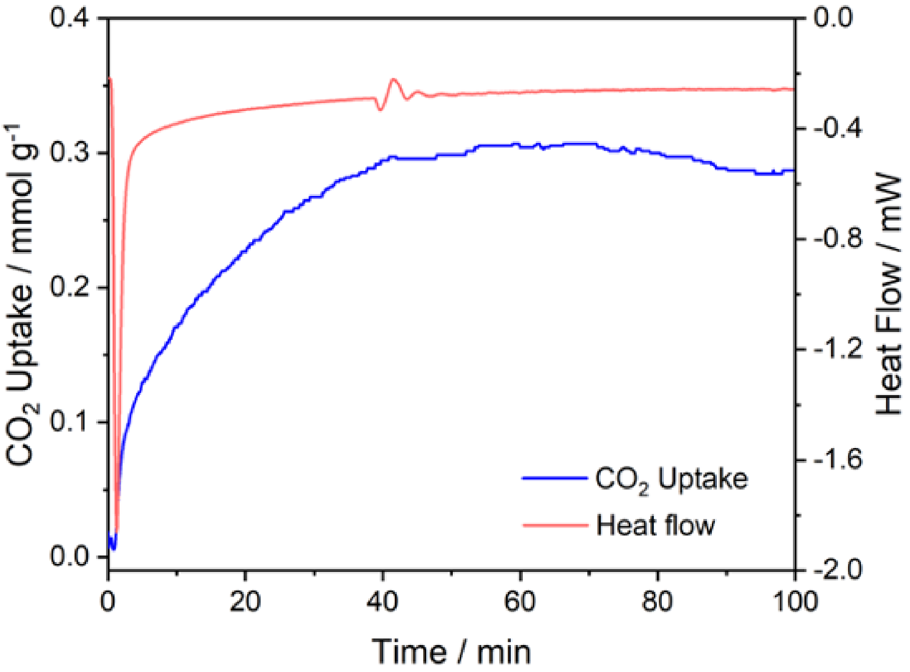

Figure S7. The CO_2 dynamic uptake profile and DSC trace of CA/MIL-101/PEI-14.5 at 25 °C.

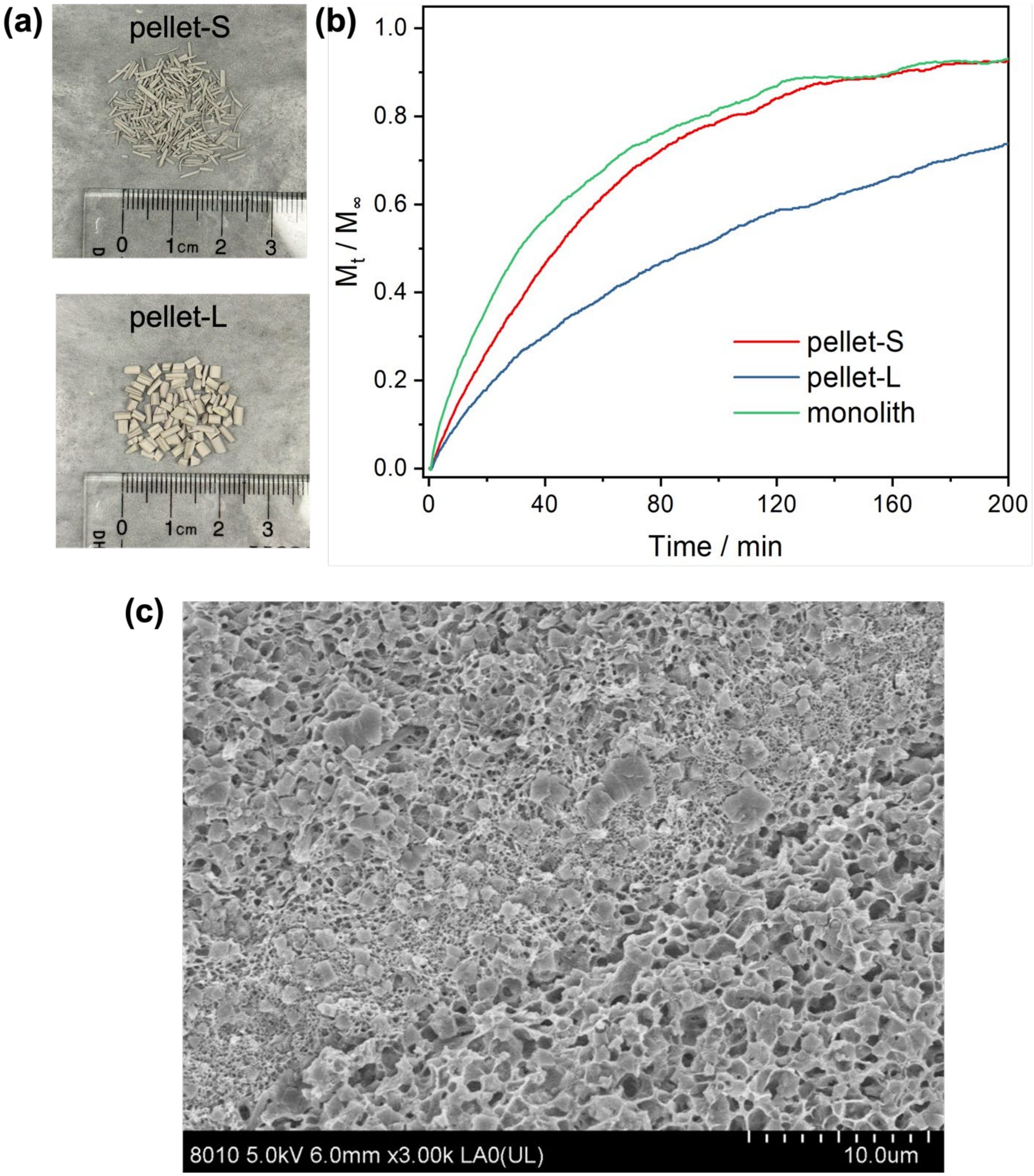

Figure S8. (a) Images of CA/MIL-101/PEI pellets. (b) Comparison of CO_2 dynamic uptake profiles of CA/MIL-101/PEI pellets and monoliths at −20 °C. These profiles were collected in the TGA/DSC system. (c) SEM image of the interface between the bottom and top 3D printed-layers of CA/MIL-101/PEI monolith.

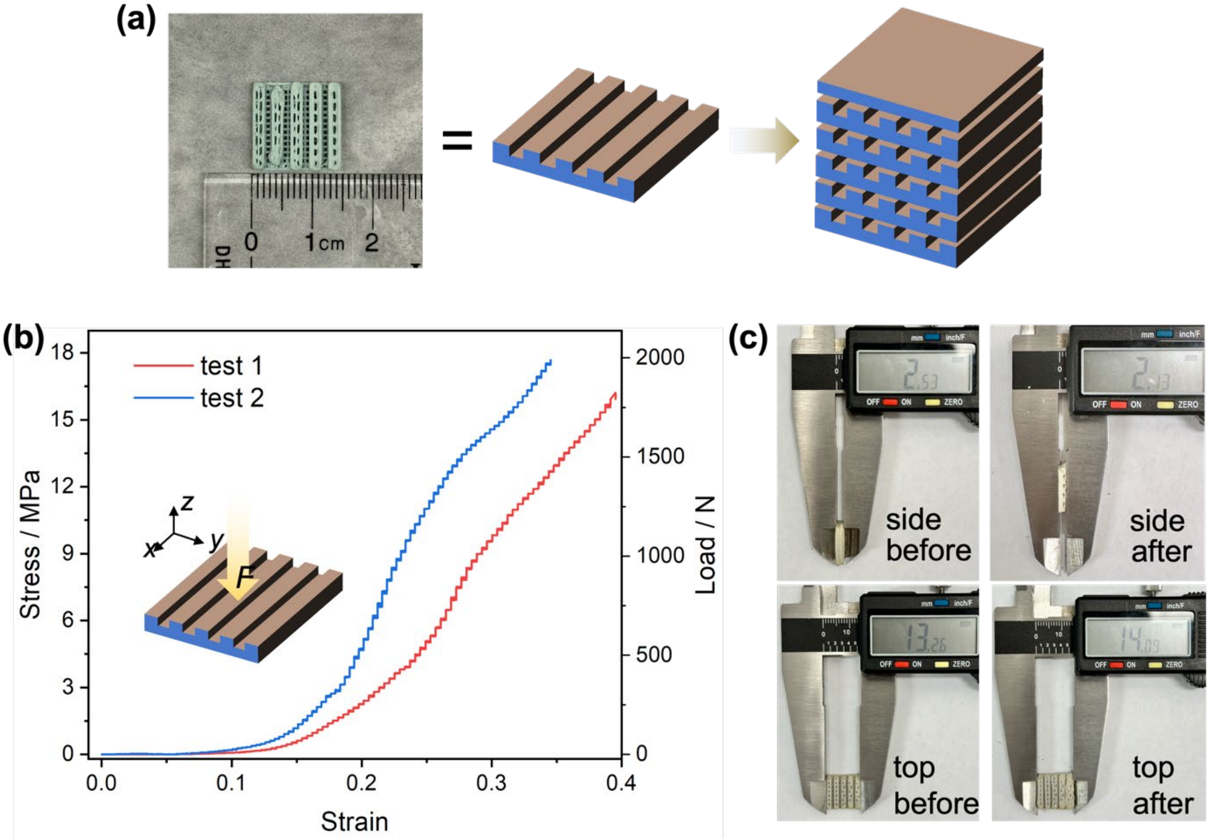

Figure S9. (a) A scheme showing the staggering of CA/MIL-101/PEI monoliths (monolith-L) for breakthrough experiments. (b) Mechanical responses of monolith-L as a function of compressive strains. The stress is calculated by the load divided by the cross-sectional area of the base of the monoliths. (c) Pictures of monolith-L before and after compression tests from the side and top perspectives.

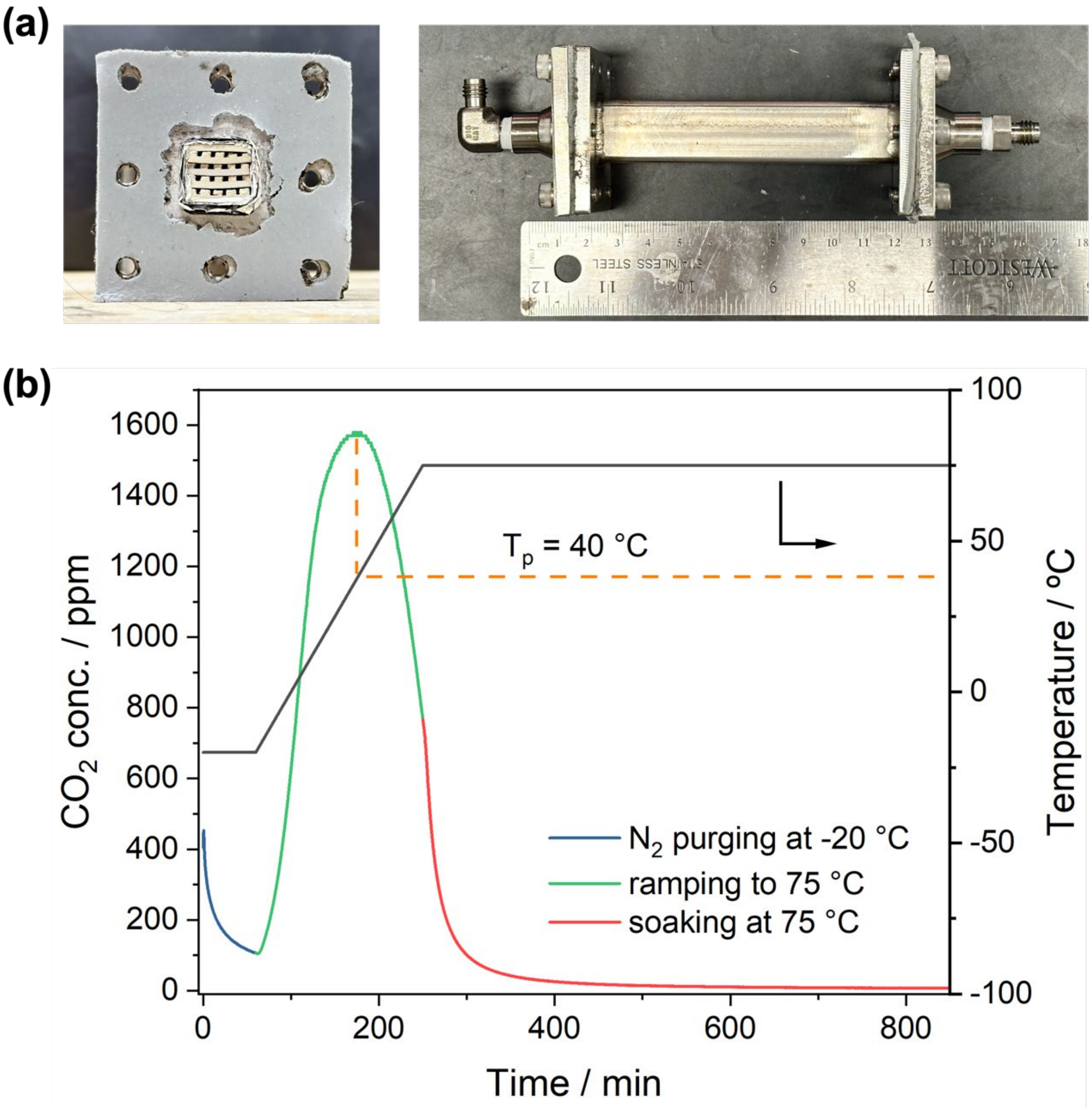

Figure S10. (a) Cross-sectional and top views of the home-made stainless-steel housing for the CA/MIL-101/PEI monoliths. (b) CO_2 temperature-programmed desorption profile of CA/MIL-101/PEI monoliths after a breakthrough experiment of dry 400 ppm CO_2 at −20 °C.

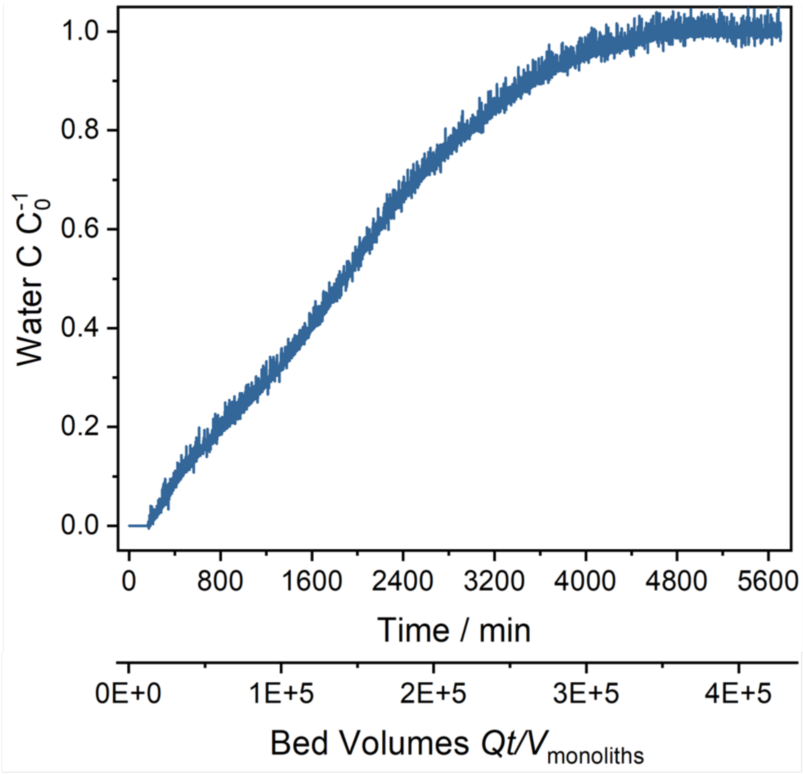

Figure S11. Water breakthrough curve during the pre-saturation step of CA/MIL-101/PEI monoliths at −20 °C using a flow rate of 200 sccm.

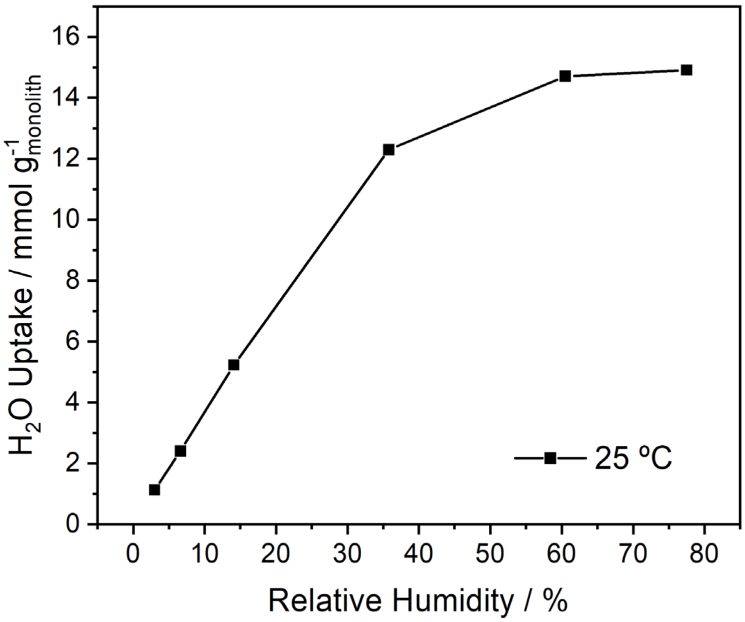

Figure S12. Water uptakes in CA/MIL-101/PEI-13.3 at 25 °C measured gravimetrically. Equilibrium time for each data point is 2 h.

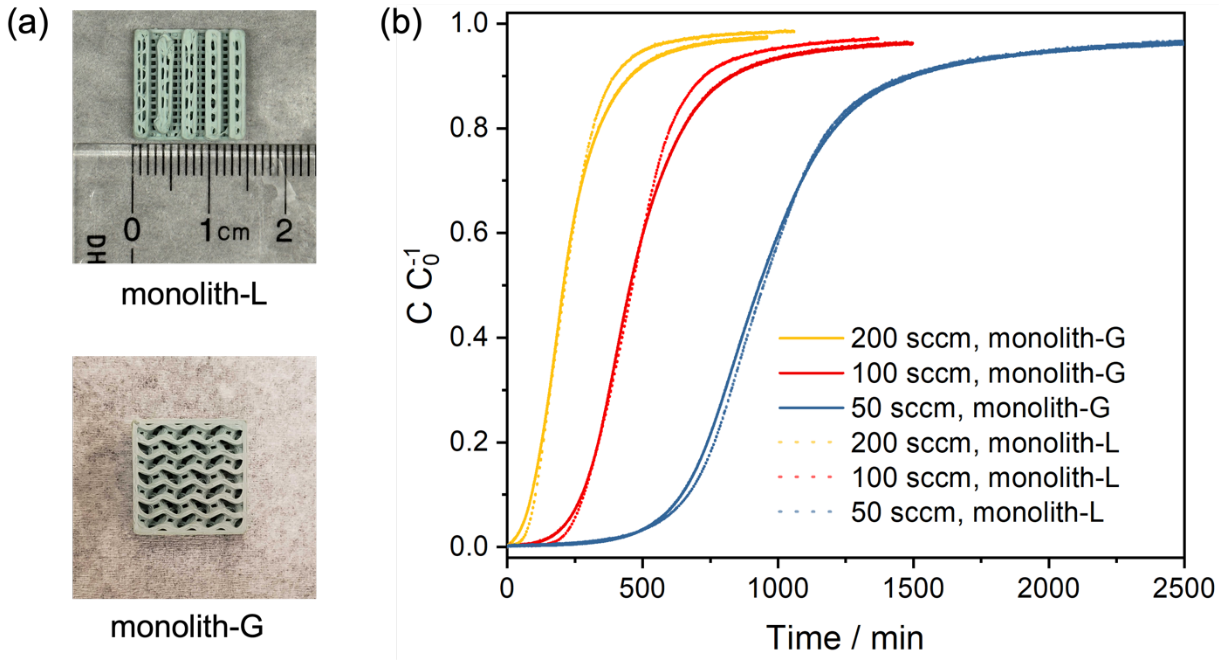

Figure S13. (a) Top views of monolith-L and monolith-G. (b) Breakthrough curves of dry 400 ppm CO_2 of fixed beds using monolith-L and monolith-G at −20 °C. Cura, the 3D printing software, was used to convert the stl file of a 15 × 15 × 4 mm monolith to G-code by (1) removing the top and bottom surfaces of the monolith; (2) setting the infill pattern to "gyroid"; and (3) setting the infill density to 25%.

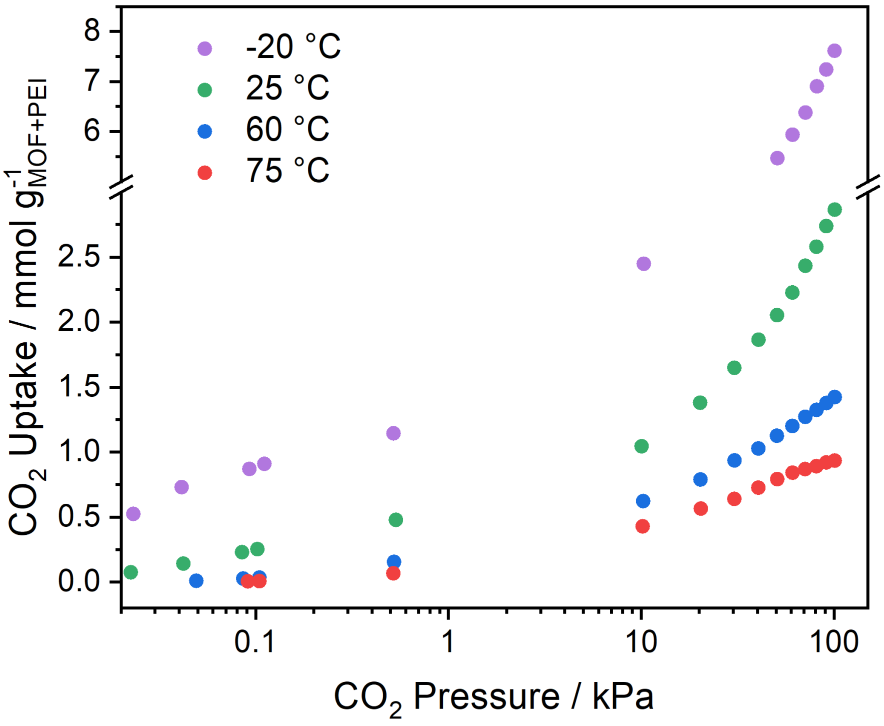

Figure S14. CO_2 uptakes in MIL-101(Cr)/PEI (10 mmol N g_{MOF}^{-1}) at different temperatures.

Table S1 Summary of CO_2 uptakes and half-adsorption time of recent DAC sorbents.

Sorbents	Form Factor	Amine Loading in Porous Support / wt%	Pseudoequilibrium Uptake at dry ~400 ppm CO_2 Condition / mmol g^{-1}	Half-Adsorption Time / min	Ref
CA/MIL-101/PEI-20.2	monolith	46	1.20 at −20 °C	32 at −20 °C	This work
CA/MIL-101/PEI-14.2	monolith	38	1.06 at −20 °C	28 at −20 °C	This work
CA/MIL-101/PEI-14.2	monolith	38	0.60 at 25 °C	7.5 at 25 °C	This work
SBA-15 supported diethanolamine and PEI S/D1/P1	powder	50	1.62 at 25 °C	25 at 25 °C	3
SBA-15 supported PEI	powder	50	1.35 at 25 °C	26.6 at 25 °C	4
SBA-15 supported TEPA	powder	50	2.3 at 25 °C	43.9 at 25 °C	4
SBA-15 supported PPI (M_w: 1000 g mol^{-1})	powder	50	1.1 at 35 °C	34 at 35 °C	5
SBA-15 supported PPI (M_w: 6700 g mol^{-1})	powder	50	0.91 at 35 °C	41.2 at 35 °C	5
Aluminum monolith	monolith	30.5	0.75 - 0.9 at 30 °C	21.7 at 30 °C	6
NPEI-SIPs	powder	49	1.1 at 25 °C	210 at 25 °C	7
AEAPDMS-NFC-FD	hydrogel	-	1.38 at 25 °C	91.8 at 25 °C	8
MIL-101(Cr) supported PEI	powder	50	1.67 at 25 °C	51 at 25 °C	2
MIL-101(Cr) supported PEI	powder	30	0.29 at 25 °C	7.5 at 25 °C	2
MIL-101(Cr)-supported TEPA	powder	50	2.19 at 25 °C	55 at 25 °C	2
MIL-101(Cr)-supported TEPA	powder	30	0.36 at 25 °C	12 at 25 °C	2
MIL-101(Cr) supported PEI	powder	50	1.85 at −20 °C	62 at −20 °C	2
MIL-101(Cr) supported PEI	powder	30	1.18 at −20 °C	34 at −20 °C	2
MIL-101(Cr)-supported TEPA	powder	50	1.59 at −20 °C	64 at −20 °C	2
HS-TEPA-50	powder	50	3.0 at 30 °C	88 at 30 °C	9

MIL-101(Cr)-supported TEPA	powder	30	1.32 at −20 °C	34 at −20 °C	2
PEI–$Mg_{0.55}$Al–O	powder	50	1.6 at 25 °C	41 at 25 °C	10
PEI- ePTFE/silica	laminate	50	1.46 at 35 °C	31 at 35 °C	11
PEI- ePTFE/silica	laminate	33	1.12 at 35 °C	13 at 35 °C	11

References

(1) Khoramzadeh, E.; Mofarahi, M.; Lee, C.-H. Equilibrium Adsorption Study of CO_2 and N_2 on Synthesized Zeolites 13X, 4A, 5A, and Beta. *J. Chem. Eng. Data* **2019**, *64*, 5648-5664. 10.1021/acs.jced.9b00690

(2) Rim, G.; Kong, F.; Song, M.; Rosu, C.; Priyadarshini, P.; Lively, R. P.; Jones, C. W. Sub-Ambient Temperature Direct Air Capture of CO_2 using Amine-Impregnated MIL-101(Cr) Enables Ambient Temperature CO_2 Recovery. *JACS Au* **2022**, *2*, 380-393. 10.1021/jacsau.1c00414

(3) Miao, Y.; Wang, Y.; Ge, B.; He, Z.; Zhu, X.; Li, J.; Liu, S.; Yu, L. Mixed Diethanolamine and Polyethyleneimine with Enhanced CO_2 Capture Capacity from Air. *Adv. Sci.* **2023**, *10*, 2207253. https://doi.org/10.1002/advs.202207253

(4) Miao, Y.; He, Z.; Zhu, X.; Izikowitz, D.; Li, J. Operating Temperatures Affect Direct Air Capture of CO_2 in Polyamine-loaded Mesoporous Silica. *Chem. Eng. J.* **2021**, *426*, 131875. https://doi.org/10.1016/j.cej.2021.131875

(5) Pang, S. H.; Lively, R. P.; Jones, C. W. Oxidatively-Stable Linear Poly(propylenimine)-Containing Adsorbents for CO_2 Capture from Ultradilute Streams. *ChemSusChem* **2018**, *11*, 2628-2637. https://doi.org/10.1002/cssc.201800438

(6) Sakwa-Novak, M. A.; Yoo, C.-J.; Tan, S.; Rashidi, F.; Jones, C. W. Poly(ethylenimine)-Functionalized Monolithic Alumina Honeycomb Adsorbents for CO_2 Capture from Air. *ChemSusChem* **2016**, *9*, 1859-1868. https://doi.org/10.1002/cssc.201600404

(7) Rim, G.; Feric, T. G.; Moore, T.; Park, A.-H. A. Solvent Impregnated Polymers Loaded with Liquid-Like Nanoparticle Organic Hybrid Materials for Enhanced Kinetics of Direct Air

Capture and Point Source CO_2 Capture. *Adv. Funct. Mater.* **2021**, *31*, 2010047. 10.1002/adfm.202010047

(8) Gebald, C.; Wurzbacher, J. A.; Tingaut, P.; Zimmermann, T.; Steinfeld, A. Amine-Based Nanofibrillated Cellulose As Adsorbent for CO_2 Capture from Air. *Environ. Sci. Technol.* **2011**, *45*, 9101-9108. 10.1021/es202223p

(9) Kulkarni, V.; Panda, D.; Singh, S. K. Direct Air Capture of CO_2 over Amine-Modified Hierarchical Silica. *Ind. Eng. Chem. Res.* **2023**, *62*, 3800-3811. 10.1021/acs.iecr.2c02268

(10) Zhu, X.; Ge, T.; Yang, F.; Lyu, M.; Chen, C.; O'Hare, D.; Wang, R. Efficient CO2 capture from ambient air with amine-functionalized Mg–Al mixed metal oxides. *J. Mater. Chem. A* **2020**, *8*, 16421-16428. 10.1039/D0TA05079B

(11) Min, Y. J.; Ganesan, A.; Realff, M. J.; Jones, C. W. Direct Air Capture of CO_2 Using Poly(ethyleneimine)-Functionalized Expanded Poly(tetrafluoroethylene)/Silica Composite Structured Sorbents. *ACS Appl. Mater. Interfaces* **2022**, *14*, 40992-41002. 10.1021/acsami.2c11143
